# Short-chain aurachin D derivatives are selective inhibitors of *E. coli* cytochrome *bd*-I and *bd*-II oxidases

**DOI:** 10.1038/s41598-021-03288-7

**Published:** 2021-12-13

**Authors:** Melanie Radloff, Isam Elamri, Tamara N. Grund, Luca F. Witte, Katharina F. Hohmann, Sayaka Nakagaki, Hojjat G. Goojani, Hamid Nasiri, Hideto Miyoshi, Dirk Bald, Hao Xie, Junshi Sakamoto, Harald Schwalbe, Schara Safarian

**Affiliations:** 1 Department of Molecular Membrane Biology, Max Planck Institute of Biophysics, Max-von-Laue-Straße 3, 60438 Frankfurt am Main, Germany; 2 Center for Biomolecular Magnetic Resonance, Institute of Organic Chemistry and Chemical Biology, Goethe-University Frankfurt Am Main, Max-von Laue-Straße 7, 60438 Frankfurt am Main, Germany; 3 Department of Molecular Cell Biology, Amsterdam Institute of Molecular and Life Sciences, Faculty of Sciences, Vrije Universiteit Amsterdam, De Boelelaan 1108, 1081 HZ Amsterdam, The Netherlands; 4 Department of Cellular Microbiology, University Hohenheim, 70599 Stuttgart, Germany; 5 Division of Applied Life Sciences, Graduate School of Agriculture, Kyoto University, Kyoto, 606-8502 Japan; 6 Department of Bioscience and Bioinformatics, Kyushu Institute of Technology, Kawazu 680-4, Iizuka, Fukuoka-ken 820-8502 Japan

**Keywords:** Biochemistry, Chemical biology, Microbiology, Molecular biology

## Abstract

Cytochrome *bd*-type oxidases play a crucial role for survival of pathogenic bacteria during infection and proliferation. This role and the fact that there are no homologues in the mitochondrial respiratory chain qualify cytochrome *bd* as a potential antimicrobial target. However, few *bd* oxidase selective inhibitors have been described so far. In this report, inhibitory effects of Aurachin C (AurC-type) and new Aurachin D (AurD-type) derivatives on oxygen reductase activity of isolated terminal *bd*-I, *bd*-II and *bo*_3_ oxidases from *Escherichia coli* were potentiometrically measured using a Clark-type electrode. We synthesized long- (C10, decyl or longer) and short-chain (C4, butyl to C8, octyl) AurD-type compounds and tested this set of molecules towards their selectivity and potency. We confirmed strong inhibition of all three terminal oxidases for AurC-type compounds, whereas the 4(1H)-quinolone scaffold of AurD-type compounds mainly inhibits *bd*-type oxidases. We assessed a direct effect of chain length on inhibition activity with highest potency and selectivity observed for heptyl AurD-type derivatives. While Aurachin C and Aurachin D are widely considered as selective inhibitors for terminal oxidases, their structure–activity relationship is incompletely understood. This work fills this gap and illustrates how structural differences of Aurachin derivatives determine inhibitory potency and selectivity for *bd*-type oxidases of *E. coli.*

## Introduction

Aurachins are isoprenoid quinoline alkaloids that have first been isolated from myxobacteria over 30 years ago^1^. These natural compounds have been sub-classified according to their structures (A-H, E, RE, SS). Previous studies have provided insights into the biosynthesis of aurachins and new methods of chemical semi- or full synthesis of aurachins have been described^2–7^. Studies on biological activity of these compounds demonstrated antiplasmodial, antifungal and antibacterial effects of aurachins. Antibacterial activity was mainly observed for gram-positive strains, like *B. subtilis* with reported minimum inhibitory concentration values ranging from 0.15 µg/ml for AurC /D to 5 µg/ml for AurA. Treatment with aurachins showed only weak inhibitory effect on gram-negative species^1^. Interestingly, experiments with the multidrug efflux transporter deficient TolC strain of *Escherichia coli* (*E. coli*) showed a moderate antibacterial effect of AurD (farnesyl) and two derivative variants with geranyl and prenyl isoprenoid side chain. These results raised hopes that this group of compounds may serve as a new antibiotic class in the future^4^. In contrast, studies on human U-2 OS osteosarcoma cells revealed that the mitochondrial membrane potential was reduced to 50–80% upon treatment with AurD and some, but not all tested, derivatives. Noticeable cytotoxicity on murine fibroblast, human carcinoma cell lines and vero cells (L929; HCT-116; K562) has been described in the same study^4^. It is conceivable that cytotoxicity is a result of an inhibitory effect on the mitochondrial NADH-ubiquinone oxidoreductase (Complex I)^1,2,4^. Thus, this kind of long-chain aurachins are not yet ready to be used as antibiotics. At the same time, the use of quinol-like species offer the possibility to gain deeper insights into the mechanism of action of respiratory quinol: O_2_ oxidoreductases. *E. coli* encodes for three terminal membrane-integrated oxygen reductases: cytochromes *bd*-I, *bd*-II and *bo*_3_. All of these terminal oxidases contribute to the generation of an electrochemical proton gradient (*proton motive force*) across the cytoplasmic membrane which is the driving force for ATP synthesis of and secondary active transport. This proton gradient is generated by either pumping proton across the membrane by cytochrome *bo*_3_ or vectorial charge separation via the cytochrome *bd* oxidase. The terminal oxidases of *E. coli* oxidize quinols in the cytoplasmic membrane and reduce oxygen to water as part of the aerobic respiration. *E. coli* is a facultative anaerobic organism that is able to adapt to different environmental oxygen conditions. In highly oxygenated environments cytochrome *bo*_3_ oxidase is the predominantly active terminal oxidase^8–11^. Cytochrome *bo*_3_ belongs to the heme-copper containing oxidase (HCO) superfamily with homologues that are widely distributed among bacteria and shares structural homology to mitochondrial oxidases. However, no eukaryotic homologues exist for the members of the cytochrome *bd* family. Cytochrome *bd*-I and *bd*-II are two additional terminal oxidases of *E. coli* with high oxygen affinity. At lower oxygen concentrations the efficiency of cytochrome *bo*_3_ decreases due to its lower oxygen affinity. To enable aerobic growth under microaerobic conditions, oxygen reduction is shifted from cytochrome *bo*_3_ to the *bd*-I and *bd*-II oxidases^12–14^. For *E. coli* it was shown that content and ratio of primarily three different quinol species adapt to environmental conditions. Ubiquinol is the main species under aerobic growth conditions. Menaquinol and demethylmenaquinol are found to be mainly present under microaerobic or anaerobic conditions^15,16^. Consequently, aurachins have been used in functional and structural studies of *bd*-type oxidases, due to their selectivity and good activity^17,18^. Interaction studies of decyl-aurachin D with cytochrome *bd* from *Azotobacter vinelandii* revealed that decyl-aurachin D acts in the vicinity of heme *b*-558 and prevents the oxidation of substrate quinols and the resulting electron transfer to heme *b-558*. Early studies with monoclonal antibodies identified the Q-loop as the plausible ubiquinol binding site^19^. Studies involving site directed mutagenesis within this region led to further characterization of the involved residues, identifying Lys252 and Glu257 to play a key role in quinol oxidation. Binding of the aurachin derivative AD5-10 (general nomenclature: first number refers to residue at position R^1^, second number refers to the residue at position R^2^) results in decrease of oxygen reductase activity in the wild-type control in accordance with spectral shifts only for heme *b-558*. Oxidase activity of some mutants remained high upon AD5-10 addition, indicating resistance to the inhibitor^20^. Hydrogen/deuterium exchange mass spectrometry revealed that AD3-11 reduces flexibility of the N-terminal Q-loop^21^. However, no other interactions were found for AD3-11, implying that no secondary binding site other than the Q-loop is present in *bd*-I for this type of compounds.

The biological activity of naturally occurring Aurachins C, D^1,2,4,22^, RE^5^, SS^6^ and P^23^ have been described previously. However, there are no systematic studies of the inhibitory potentials of short-chain AurD analogues on neither cytochrome *bd* oxidase, nor on cytochrome *bo*_3_ oxidase from *E. coli*. We expand the variety of inhibitors by introducing new side chains to the aurachin scaffold to obtain a more comprehensive understanding of potential cytochrome *bd* oxidase inhibitors. Functional assays on purified protein with a diverse set of quinolones enabled us to identify structural features that determine selectivity and potency.

## Results and discussion

**Figure 1:**
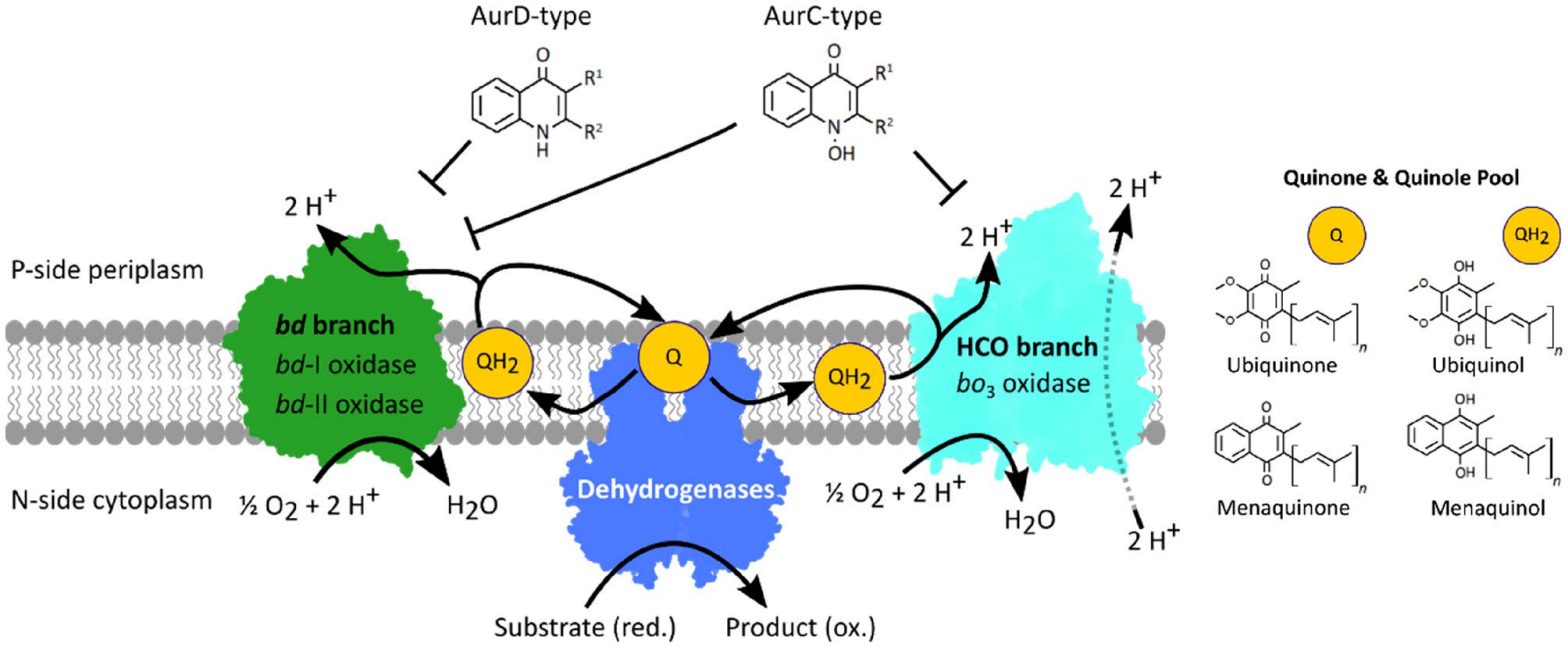
Schematic representation of the two terminal oxidase branches of *E. coli* composed of the proton pumping (HCO) type cytochrome *bo*_3_ oxidase and two *bd*-type oxidases. All three enzymes oxidize membrane quinols (QH_2_) and transfer electrons to their respective active site for the reduction of molecular oxygen to water. The resulting quinones (Q) are reduced by transfer of two electrons in the enzymatic action of dehydrogenases. Our inhibitor design is based on two scaffolds where AurD-type compounds are expected to inhibit only *bd* branch enzymes and AurC-type compounds are non-selective on *bd* and HCO branch/terminal oxidases. Green: *bd*-oxidases; Turquoise: *bo*_3_ oxidase; Blue: Dehydrogenases.

We synthesized a new set of short-chain AurD-type compounds as derivatives of the previously characterized natural source inhibitor AurD. AurD has a similar molecular scaffold as the substrate quinol species of quinol oxidases: demethylmenaquinol-8 (DMK-8) and menaquinol-8 (MK-8). In *E. coli* DMK-8 and MK-8 are present under microaerobic and anaerobic conditions. However, ubiquinols (UQ) are the main species in an aerobic environment^24^. In contrast to UQ species that contain a benzoquinone framework, MK-8 possesses a naphthalene 1,4-dione ring with a methyl-group and an eight-unit isoprene unit side chain. The main difference between MK-8 and aurachins, as indicated in Fig. 1, is the molecular scaffold. Aurachins consist of quinolone heterocycles with an N–OH (AurC) or N–H (AurD) group at position 1. Originally, AurC and AurD possess a methyl and an isoprenoid side chain. This structural similarity inspired us to synthesize and test a set of aurachin analogues with short aliphatic side chains (C4, butyl to C8, octyl) or long aliphatic side chains (C10, decyl or longer) at position R^1^. One promising candidate 2-(2-Heptyl)-3-methyl-4(1H)-quinolone (AD7-1) was modified further to examine the influence of structural changes on its inhibitory effect. Therefore, we included two more structures: unsaturated AD7-1* with a double-bond in the heptyl-side chain and AD7-2 which includes an elongated ethyl side chain at R^2^ in addition to the heptyl group at R^1^.

### Synthesis

The 4-quinolone scaffold of aurachins forms the basis of numerous natural products and drugs. Therefore, several synthetic methods can be found in the literature that offer a way for construction of such substituted heterocycles. An example is the nickel catalyzed cycloaddition of isatoic anhydride through addition of reactive alkyne derivatives^25^. However, this method is more suitable for symmetrical alkynes due to the difficult purification of delivered regioisomers in case of unsymmetrical alkynes. In 2020, Liu et al. reported a metal free synthetic strategy that combined Michael addition and Smiles rearrangement generating 1,2,3-trisubstituted 4-quinolones^26^. The feasibility of this base-mediated sequential protocol is limited to the use of N-arylated sulfonamide. In contrast, *Dejon *et al*.* presented an efficient possibility based on Conrad-Limpach cyclization of several isoprenoid side chain substituted enamine derivatives^27^. By employing this simplified procedure, an ensemble of 3-alkyl substituted 2-methyl-4-quinolones with different aliphatic side chain length were successfully synthesized. Since not every desired allylic bromide was commercially available, we started with the halogenation of appropriate allylic alcohols that was performed by following a method previously described in the study of Camps et al.^28^*.* In this synthesis strategy, the allylic alcohol derivative firstly undergoes a conversion to the corresponding mesylate that reacted with Lithium bromide yielding the allylic bromide. Subsequently, ethyl acetoacetate was selectively substituted with previously obtained allylic bromides at position R^2^ by using sodium hydride (NaH) in tetrahydrofuran (THF). In a next step, the ceric ammonium nitrate (CAN) catalyzed enamination with aniline of synthesized 2-substituted ethyl acetoacetate intermediate was carried out. Notably, this method afforded only moderate yields of the desired β-enaminones. Improving catalytic activity of CAN by solvent change was not successful. A putative reason for this can be found in steric hindrance caused by the aliphatic side chain. However, the advantage was that β-enaminones could be utilized as crude product in the final Conrad–Limpach cyclization step to obtain substituted 2-methyl-4-quinolones. These were purified by chromatography on a silica gel column employing a cyclohexane/ethyl acetate gradient to remove diphenyl ether residues. For further details, please refer to the Supplementary information.

**Figure 2:**
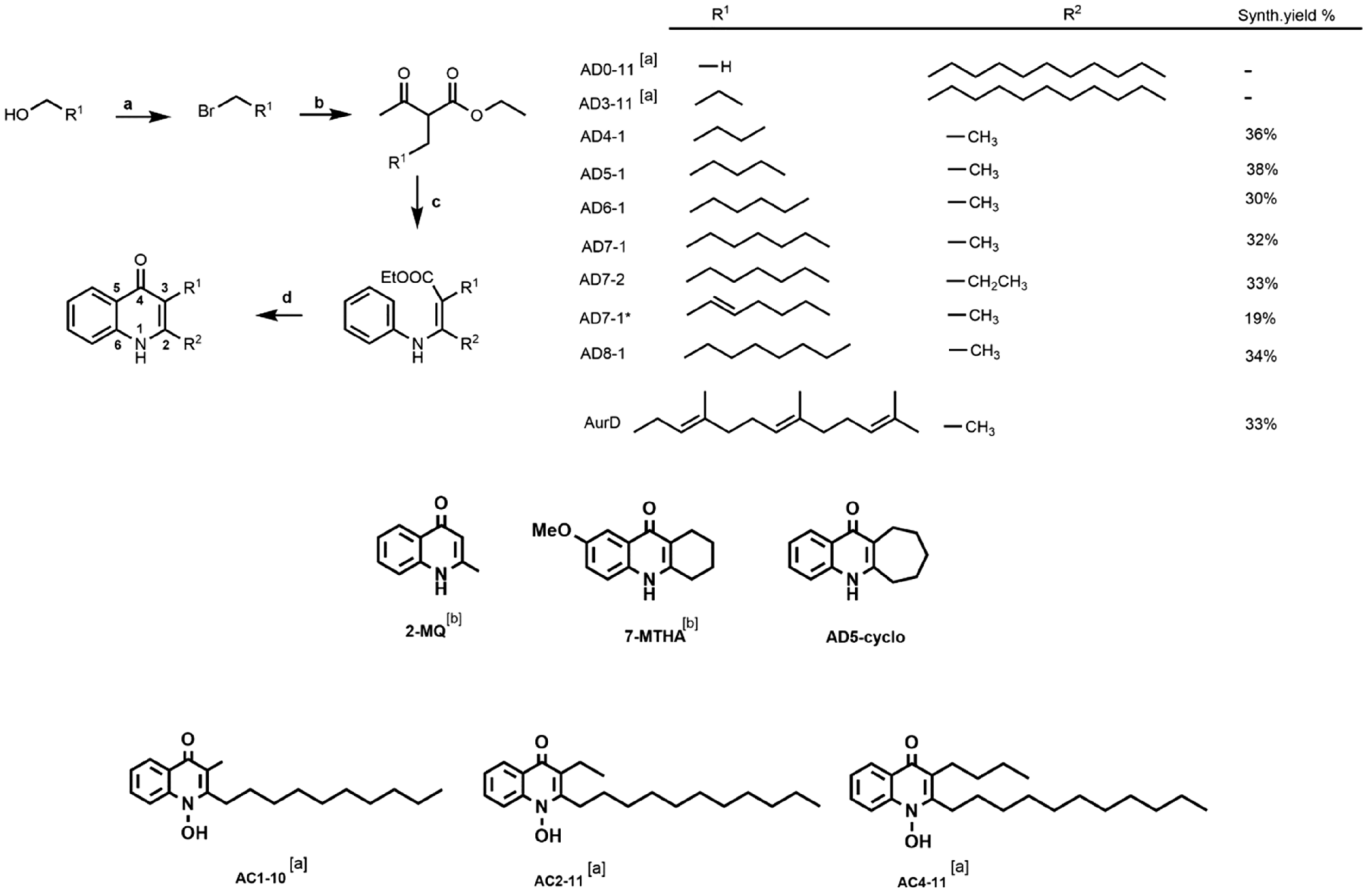
Overview of test compounds. General synthesis route to generate substituted 2-methyl-4-quinolones (AurD-type). (**a**) Allyl alcohol (1.0 equiv.), Mesyl chloride (1.3 equiv.), NEt_3_ (2.0 equiv.), LiBr (4.0 equiv.), anh.THF, − 40°, − 0 °C, 1 h, 88%. (**b**) Allyl bromide (1.1 equiv.), NaH_60%_(1.1 equiv.), Ethyl acetoacetate (1.0 equiv.), anh. THF, 0°-RT, 12 h, 54%. (**c**) 2-Allylacetoacetate (10.0 equiv.), CAN (0.5 equiv.), Aniline (10.0 equiv.), anh. EtOH, RT, 2 h, 23%. (**d**) Enamine (1.0 equiv.), diphenyl ether, 250 °C-RT, 1 h, 33%. Reaction yields correspond to the average of all synthesized derivatives. [a] from^17^ [b] 2-methylquinolin-4-ol (2-MQ) and 7-methoxy-1,3,4,10-tetrahydro-9(2H)-acridinone (7-MTHA) are commercially available. *Indicates double bond in position C1 of R^1^.

In addition to the synthesized short-chain aurachin derivatives, we tested one compound with a cycloheptanyl cycle (AD5-cyclo). We included two commercially available substances: 7-methoxy-1,3,4,10-tetrahydro-9(2H)-acridinone (7-MTHA) as a tri-cyclic representative with cyclohexanyl ring in combination with a methoxy group at position 7 and 2-methyl-4-quinolinol (2-MQ) as a smaller AurD-type representative with only one methyl side chain at position R^2^. In order to provide a comprehensive overview on the relationship between inhibitory potential and chemical structure, we included the natural source compound AurD, two long-chain AurD-type and three long-chain AurC-type compounds in our test set. Inhibitory effects of the latter compounds were previously characterized for the cytochromes *bd*-I and *bo*_3_ oxidases from *E. coli*^17^. Further, we used 2-heptyl-4-quinolinol 1-oxide (HQNO) as a well described reference inhibitor^22,29,30^. An overview of the test set is given in Fig. 2.

### Oxygen reductase assay to determine inhibition activity

**Figure 3:**
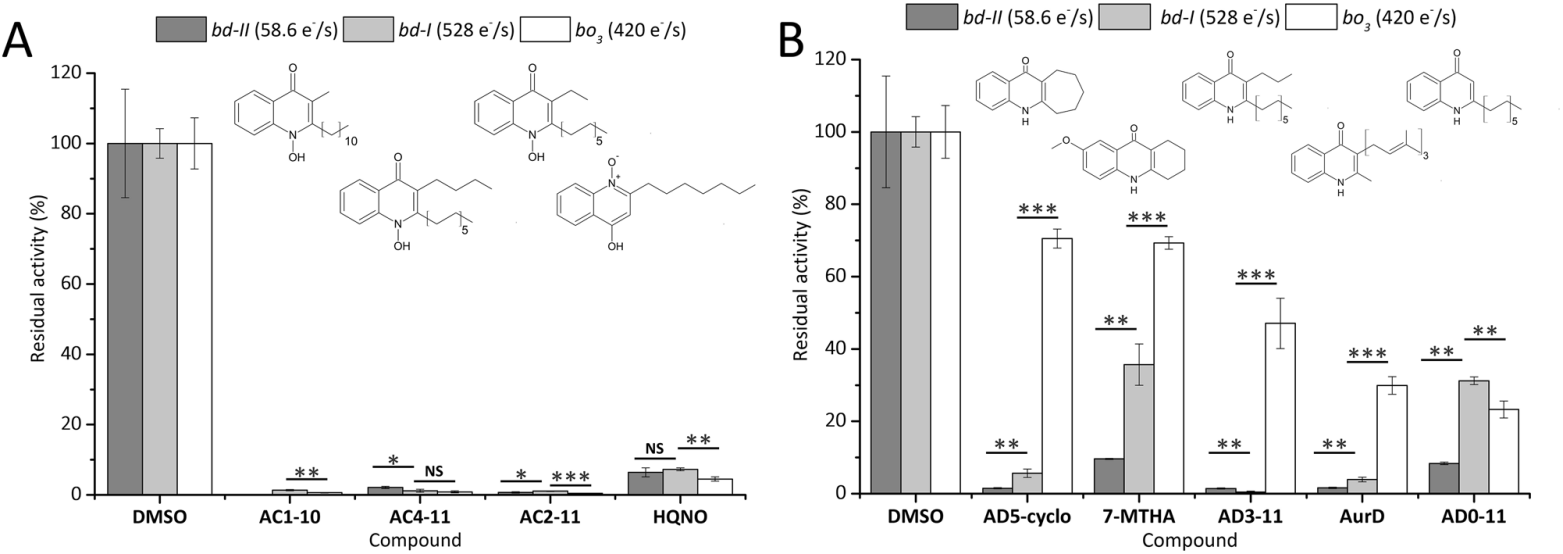
(**A**) Screening results for cytochromes *bd*-I (light-grey), *bd*-II (dark-grey) and *bo*_3_ (white) in presence of AurC-type compounds and HQNO. (**B**) Screening results for cytochromes *bd*-I, *bd*-II and *bo*_3_ in presence of cyclic and long-chain AurD-type compounds. Inhibition assay using test compounds at 250 µM in presence of 200 µM ubiquinone-1 and 5 mM dithiothreitol. Inhibitory activities were calculated from oxygen consumption rates at RT. Reference activity (100%) of each oxidase was determined in presence of DMSO to exclude secondary effects of the solvent. Data are given as mean ± S.E.M. (n = 3) ***p < 0.001, **p < 0.01, *p < 0.05, ^NS^*p* > 0.05.

**Figure 4:**
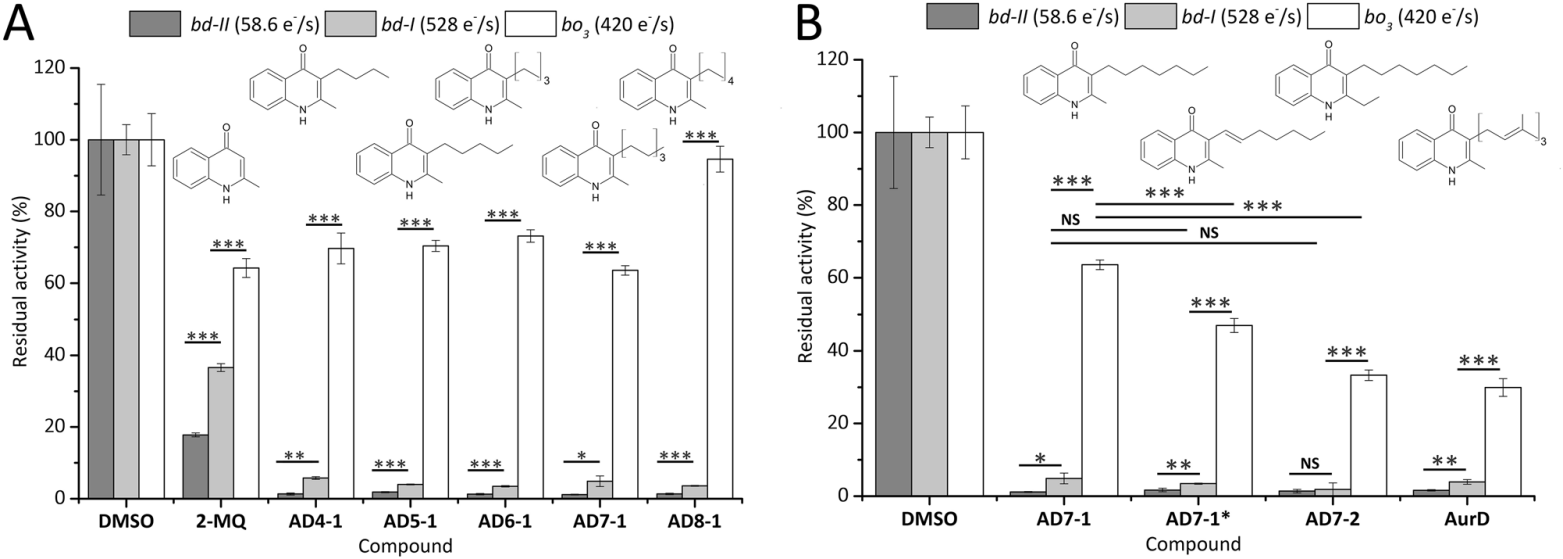
(**A**) Screening results for cytochromes *bd*-I (light-grey), *bd*-II (dark-grey) and *bo*_3_ (white) for short-chain AurD-type compounds. (**B**) Screening results for selected AurD-type compounds with decreasing residual activity for cytochrome *bo*_3_ from left to right. Inhibition assay using test compounds at 250 µM in presence of 200 µM ubiquinone-1 and 5 mM dithiothreitol. Oxygen reduction activity was calculated from oxygen consumption rates at RT. Reference activity (100%) of each oxidase was determined in presence of DMSO to exclude secondary effects of the solvent. Presented data are mean ± S.E.M. (n = 3) ***p < 0.001, **p < 0.01, *p < 0.05, ^NS^*p* > 0.05.

To determine inhibition of our test set compounds and to assess the selectivity for *bd*-type oxidases we tested the inhibitory effect via potentiometric determination of oxygen reduction using purified protein samples and ubiquinol-1 as electron donor. In this set-up, we kept the compound at a high molar excess compared to the protein. Figures 3 and 4 illustrate the screening results for cytochromes *bd*-I, *bd*-II and *bo*_3_, respectively. A comparative overview of all compounds is given in SI Table S1. All tested AurC-type compounds have a strong inhibitory effect on cytochromes *bd*-I*, bd*-II and *bo*_3_ resulting in residual activity < 3% at 250 µM concentration. Despite a strong inhibitory effect, none of the tested AurC-type compounds selectively inhibits a certain terminal oxidase (Fig. 3A). Among these compounds, AC2-11 has the strongest inhibitory effect on all three oxidases. These derivatives show a higher inhibitory activity compared to the reference inhibitor HQNO. HQNO is a non-selective ubiquinol oxidase inhibitor that acts on cytochromes *bo*_3_ and *bd* at low micromolar concentrations*.* For AurC and its analogues it was reported that replacement of N–OH with an N–H group decreases the inhibitory potential only for cytochrome *bo*_3_, keeping a strong inhibitory effect on cytochrome *bd*^17^. Correspondingly, we observed a clear inhibition of cytochromes *bd*-I and *bd*-II with all AurD-type compounds. Regarding the long-chain derivatives AC4-11, AC2-11 and AD3-11, inhibition of cytochromes *bd*-I and *bd*-II and *bo*_3_ highlight the importance of the residue at the nitrogen group at position 1 for enzyme selectivity. AD3-11 reduces activity of both *bd*-type oxidases but is less active on cytochrome *bo*_3_ (Fig. 3B). However, AD3-11 shows the highest inhibition potential of cytochrome *bd*-I. In case of cytochrome *bo*_3_ an inhibitory effect of only ~ 50% at 250 µM was reached with AD3-11. The AurD-type compound AD0-11 marks an exception, as we observed a comparatively low residual activity of 23% for cytochrome *bo*_3_ in presence of this compound. Comparison with AD3-11, that possesses an additional propyl side chain, indicates that elongation of the hydrocarbon side chain at position R^1^ reduces the inhibitory potential for cytochrome *bo*_3_. Regarding the cyclic derivatives, 7-MTHA causes only a minor inhibition of cytochrome *bd*-I*.* This effect could be due to the methoxy group in position 7 that introduces an additional polarity to the molecule. Steric hindrance as a consequence of the third ring structure is less likely, because AD5-cyclo is bulkier and reduces activity < 10% of cytochromes *bd*-I and *bd*-II.

All tested short-chain AurD-type compounds clearly showed inhibition of cytochromes *bd*-I and *bd*-II resulting in residual activities for *bd*-I of < 10% and *bd*-II of < 2% at 250 µM compound concentrations (Fig. 4). The new AurD-type compounds reduce cytochromes *bd*-I and *bd*-II activities in a higher extend compared to HQNO. Interestingly, increasing heptyl to octyl resulted in a higher residual activity of cytochrome *bo*_3_. This observation suggests a higher selectivity for cytochromes *bd*-I and *bd*-II. AD7-1, AD7-1* and AD7-2 have a similar inhibitory effect compared to AurD on both *bd-type* oxidases (Fig. 4B). However, for cytochrome *bo*_3_ we determined 30% residual activity in the presence of 250 µM AurD. Hence, AurD is the least selective inhibitor regarding the HCO-type *bo*_3_ oxidase within our test set. We observed a decreased inhibitory effect for compounds without a second chain at position R^2^. The small AurD-type compound 2-MQ lacks the second aliphatic residue and is one of the least active compounds. In presence of 2-MQ and AD0-11 cytochrome *bd*-I possesses more than 30% residual activity, indicating that the methyl side chain at R^1^ is important for the inhibitory potential (Fig. 3B). In order to find new cytochrome *bd* selective inhibitors, these findings motivated us to concentrate on short-chain compounds that do not inhibit cytochrome *bo*_3_ as strongly as the *bd* oxidases.

We investigated cytochrome *bd*-I in further experiments since we found a higher inhibition effect, compared to cytochrome *bd*-II. To facilitate a ranking of our test set we determined *K*_i_ apparent (*K*_i_^app^) as described earlier^29,30^. The *K*_i_^app^ represents the inhibitor concentration at which the residual oxygen reductase activity of the terminal oxidase is 50%. These *K*_i_^app^ values of AurD-type compounds with respect to cytochrome *bd*-I are summarized in Table 1. As expected, 2-MQ, 7-MTHA and AD0-11 which already had minor inhibitory effects on cytochrome *bd*-I were also the candidates with *K*_i_^app^ values in the higher micromolar range (Figs. 3, 4). This finding supports our hypothesis that the methyl group at R^1^ is important for inhibition of *bd*-I oxidase*.* Reduced inhibitory activity of 7-MTHA could be traced to a different molecule shape because of the third ring structure or the additional hydrophilicity obtained by the methoxy group. For AD4-1, AD5-1 and AD5-cyclo *K*_i_^app^ was found to be in the medium micromolar range. All three compounds showed good selective inhibition at high concentration for cytochromes *bd*-I and *bd*-II (Figs. 3, 4)*.* It is conceivable that the short aliphatic side chains are not able to allow sufficient interactions with the enzyme to result in a higher inhibitory effect. Additionally, as cyclic compounds AD5-cyclo and 7-MTHA showed minor inhibitory effects, it is plausible that aliphatic or isoprene-like side chains are favorable. For AD5-cyclo it is plausible that the cycloheptene group is not as suitable to interact with cytochrome *bd*-I as aliphatic or isoprene-like side chains. We suggest that the higher flexibility of aliphatic and isoprene-like side chains enables more intense interactions with the binding region and consequently results in stronger inhibition of cytochrome *bd*-I.

**Table 1:** *K*_i_^app^ values of selected AurD-type compounds acting as inhibitors of cytochrome *bd*-I from *E. coli* at a final enzyme concentration of 30 nM.

Compound	*K*_i_^app^ *bd-*I	Concentration range
2-MQ	> 250 µM	> 50 µM
7-MTHA	> 250 µM	
AD0-11	56.6 ± 5.9 µM	
AD4-1	5.4 ± 0.7 µM	1–10 µM
AD5-cyclo	4.0 ± 0.6 µM	
AD5-1	0.81 ± 0.04 µM	
AD8-1	0.66 ± 0.06 µM	0.1–1 µM
AD7-1*	0.39 ± 0.04 µM	
AD3-11	0.30 ± 0.04 µM	
AD6-1	0.28 ± 0.04 µM	
AD7-1	0.13 ± 0.02 µM	
AD7-2	0.12 ± 0.02 µM	
AurD	0.018 ± 0.002 µM	> 0.1 µM

Regarding the short-chain compounds, we observed that with increasing chain length from butyl to heptyl *K*_i_^app^ decreases. For AD7-2, *K*_i_^app^ is 45-fold lower compared to AD4-1 (120 nM vs. 5.4 µM). With respect to AurD, our synthesized short-chain AurD-type compounds result in higher *K*_i_^app^ values for cytochrome *bd*-I. However, our new set of compounds appears to have a higher selectivity towards cytochrome *bd*-I than AurD (Fig. 4). We determined *K*_i_^app^ in the nanomolar range (0.1–1 µM) for AD6-1, AD7-1, AD7-1*, AD7-2, AD8-1 and AD3-11. For AD7-1 and AD7-2 we determined lowest *K*_i_^app^ values in the nanomolar range. Compared to AD7-2, AD8-1 resulted in a sixfold increased *K*_i_^app^ value (120 nM vs. 660 nM). AD3-11 differs from AD0-11 by the presence of a propyl residue at position R^1^. This change results in a 188-fold higher inhibitory potential of AD3-11 for cytochrome *bd*-I*,* compared to AD0-11.

Because we were able to identify AD7-1 as a good selective inhibitor for cytochrome *bd*-I, we used this scaffold for further modifications. We explored how introduction of a double bond or elongation of R^2^ affects inhibitory activity (Fig. 4B). Introducing a double bond within the side chain at R^1^ did not significantly affect the inhibitory potential for cytochrome *bd*-I (AD7-1 vs. AD7-1*). Elongation of the residue in position R^2^ (AD7-1 vs. AD7-2) or introduction of a double bond within the side chain at R^1^ (AD7-1 vs. AD7-1*) resulted in a higher inhibition of cytochrome *bo*_3_. For further characterization, we determined *K*_i_^app^ for cytochrome *bo*_3_. Because of the low inhibitory effect on cytochrome *bo*_3_ by most of the tested compounds, *K*_i_^app^ determination was only possible for a part of the test set compounds. A summary of *K*_i_^app^ for cytochromes *bd*-I and *bo*_3_ is given in Table 2. Among the AurC-type compounds we found AC2-11 to be the most potent inhibitor for all tested oxidases. Moreover, our results imply that the butyl side chain in AC4-11 results in lower inhibition of the cytochrome *bd*-I activity. Miyoshi et al. described AC1-10 and AC1-11 as the most potent competitive inhibitors for cytochromes *bd* and *bo*_3_^17^. In case of AC2-11 *K*_i_^app^ for cytochromes *bo*_3_ (46 nM) and *bd* (440 nM) have been determined spectrophotometrically^17^. Their final protein concentration was 30 to 200 times lower compared to our assay conditions and a preincubation period of protein and compound was implemented^17^. Thus, we can confirm the non-selective inhibition of AC2-11 and a high potency of the long-chain AurC-type compounds in inhibition of cytochrome *bd*-I oxygen reduction activity.

**Table 2:** *K*_i_^app^ for *bd*-I and *bo*_3_ oxidoreductases for selected test compounds at a final enzyme concentration of 30 nM.

Compound	*K*_i_^app^ *bd-*I	*K*_i_^app^ *bo*_3_
AD7-1	0.13 ± 0.02 µM	> 250 µM
AD7-1*	0.40 ± 0.04 µM	> 250 µM
AD3-11	0.30 ± 0.03 µM	> 100 µM
AurD	0.018 ± 0.002 µM	> 10 µM
HQNO	3.6 ± 0.5 µM	2.6 ± 0.4 µM
AC2-11	0.034 ± 0.006 µM (0.44 µM)^[b]^	0.031 ± 0.006 µM (0.046 µM)^[b]^
AC1-10^[a]^	0.082 ± 0.011 µM (0.28 µM)^[b]^	(0.013 µM)^[b]^
AC4-11^[a]^	0.106 ± 0.013 µM (0.34 µM)^[b]^	(0.144 µM)^[b]^

[a] No data available for *bo*_3_ [b] previous data from^17^.

Regarding the AurD-type compounds, we identified AD7-1 and AD7-1* as potent inhibitors for cytochrome *bd*-I while causing no cytochrome *bo*_3_ inhibition in the low nanomolar to micromolar range (Fig. S5). For AurD we found a difference of three orders of magnitude for *K*_i_^app^_-bo3_ vs. *K*_i_^app^_-bd-I_ (< 10 µM vs. 18 nM) (Fig. S5; Table 2). For AD3-11 we determined a ratio of around 300. Therefore, it is noteworthy that the short-chain AurD-type compounds AD7-1 and AD7-1* share a similar selectivity for *bd-I* as AurD. Further we can confirm the inhibitory activity of AurC-type compounds in a nanomolar range on both different types *bd* and *bo*_3_ terminal oxidases in *E. coli*.

Short- and long-chain AurD-type compounds are reported to be efficient inhibitors of the isolated cytochrome *bc*_1_ complex (complex III) from beef heart mitochondria^31^. Similar to our findings, an increase of the inhibitory potential with increasing alkyl chain length was reported^31^. A maximum inhibitory activity was determined for 2-undecylquinolone (AD11-0, p*IC*_50_:6.66)^31^. Here, the undecyl chain in position R^2^ is switched with R^2^, compared to our AD0-11^31^. A further increase of the alkyl chain length (resp. position R^1^ in Fig. 2) results in reduced inhibition^31^. Our results show a similar trend, as the inhibition of cytochrome *bd*-I reached a maximum for AD7-1 and decreased for AD8-1. Moreover, addition of a 3-methyl group at position R^2^ further increases the potency of inhibition and results in the highest inhibition by 3-undecyl-3-methylquinolone (AD11-3, p*IC*_50_:7.70)^31^. Elongation of the 2-alkyl chain decreases the inhibition^31^. A parabolic dependence of inhibition on the chain length was observed^31^. Our results confirm an increase of inhibition by the long-chain aurachins from AD0-11 to AD3-11 for all tested oxidases. Furthermore, cytochrome *bo*_3_ was the least sensitive. Further, we could identify a similar parabolic dependence of inhibitory potency of the chain length for our compounds, as the inhibitory activity on cytochrome *bd*-I was the highest for 2-heptyl-3-methyl-quinolone (AD7-1).

## Conclusions

Specific protein inhibitors can be of use not only as antibiotically active compounds, but also as binding ligands to enable deeper investigations of protein mechanisms. As deeper investigations of the respiratory chain from *E. coli* contribute knowledge to a broad spectrum of bacteria, introducing specific inhibitors in structural investigations can open new perspectives. Earlier studies shed light on the binding site and the modes of cytochrome *bd* inhibition. However, no structure with bound aurachin has been published yet, due to lack of a defined conformation or increased flexibility of the quinol binding site. To clarify the nature of aurachin *bd* interactions, research about structure–function relationship is urgently required. Here, we investigated the selectivity and potency of AurC- and AurD-type compounds on the oxygen reduction activity of all three terminal oxidases of *E. coli* (cytochromes *bd*-I, *bd*-II and *bo*_3_). We confirmed that three AurC-type N-oxide-quinolones have a similar inhibitory potency in a nanomolar range on cytochromes *bd*-I and *bo*_3_. Two short-chain AurD-type compounds AD7-1 and AD7-1* are highly selective towards cytochrome *bd*-I with similar *K*_i_^app^ values as the natural compound AurD. In addition, we provide evidence that inhibitory activity increases with increasing chain length at position R^1^ of the 2-methyl-4-quinolones backbone. AD7-1 (2-heptyl-3-methyl-quinolone) combines properties of high inhibitory potency and selectivity for cytochrome *bd*-I*.* From the set of characterized inhibitors, AD7-1 qualifies as the best candidate to be subjected to downstream inhibition trials on a physiological level.

## Material and methods

### Chemicals

The long-chain AurD-type compounds AD3-11, AD0-11 and AD4-1 and AurC-type compounds AC1-10, AC2-11 and AC4-11 used in this study were synthesized as described previously^17^. 2-MQ and 7-MTHA were purchased from Sigma.

### Production of cytochrome *bd*-I and *bd*-II from *E. coli*

Cytochrome *bd* oxidase from *E. coli* was produced in *E. coli* C43 (DE3) Δ *bo*_3_ (CLY) cells (kindly provided by Prof. Robert B. Gennis, University of Illinois) transformed with pET17b-*cydABX-*StrepII plasmid, carrying carbenicillin/kanamycin resistance (*bd*-I) or the pET17b-*appCBX*-StrepII plasmid^32^ carrying a carbenicillin/chloramphenicol resistance (*bd*-II). 1 ml of 50% glycerol stock was added to 50 ml LB with corresponding antibiotics (50 µg/ml carbenicillin—carb; 50 mg/ml kanamycin—kan; 25 µg/ml chloramphenicol—cam) and incubated at 175 rpm at 37 °C for 8 h. Preculture was transferred to 1 L LB-carb-kan/cam to grow overnight. 2.5 L LB-kan/cam was inoculated with 70 ml overnight culture supplemented with 0.025 mM IPTG to start basal production from the beginning. After reaching OD_600_ 0.7 *bd*-I*/bd*-II oxidase production was started by adding IPTG to a final concentration of 0.25 mM. 4 h incubation at 37 °C followed by 16 h at 30 °C. Cell harvesting was carried out by centrifugation with Avanti J-26XP at 4 °C at 8000*g*. Cell disruption via microfluidizer for four cycles at 80 psi in 50 mM sodium phosphate buffer (NaPi) pH 8.0 and 100 mM NaCl supplemented with 1 mM MgCl, recombinant DNase I (Sigma) and protease inhibitor Aminoethyl-benzene-sulfonyl fluoride (Pefabloc, Roche). Low-velocity centrifugation at 5000*g* at 4 °C for 30 min before high-velocity centrifugation of the supernatant at 220,000*g* at 4 °C for 90 min. Membrane pellets were resuspended in 50 mM NaPi (pH 8.0), 100 mM NaCl containing buffer and stored at − 80 °C.

### Streptactin purification of *E. coli bd*-I and *bd*-II oxidase

Isolated membranes were solubilized in 50 mM NaPi (pH 8.0), 100 mM NaCl with 1% n-dodecyl β-d-maltoside (β-DDM) to the mass ratio of 1:5 detergent:membrane protein at 4 °C for 40 min on an orbitalshaker followed by removal of unsolubilized material by 70,000*g* for 30 min. Avidin was added to the filtrated supernatant to a final concentration of 0.2 mg/ml. Affinity chromatography was done via peristaltic pump with prepacked 5 ml StrepTrap HP column (GE Healthcare) equilibrated with 20 mM NaPi (pH 8.0), 100 mM NaCl, 0.02% DDM at a flow rate of 3 ml/min. Washing was carried out with the same buffer for 15CV. For elution this buffer was supplemented with 10 mM desthiobiotin (IBA Lifesciences). Sample dialysis with Slide-A-Lyzer (CutOff 10 K) dialysis cassettes (Thermo Fisher Scientific) in 4L 50 mM NaPi (pH 8.0), 100 mM NaCl with 1% β-DDM overnight. Presence of the *E. coli bd* type oxidase and purity of the product was analyzed by SDS-page and native page gel electrophoresis.

### Production and Ni–NTA purification of cytochrome bo_3_ from *E. coli*

Strain GO195 transformed with pIRHisA plasmid was a kind gift from by Prof. Robert B. Gennis, University of Illinois. For *bo*_3_—oxidase production a protocol from^33^ was used. Purification protocol was modified according to^33^ and described in detail in^34^*.*

### Oxygen reductase activity measurements

Oxygen reductase activity was measured as oxygen consumption rate of purified protein by OX-MR Clark-type oxygen electrode linked to a PA 2000 picoammeter and to the ADC-216 AD-converter. Data recording with SensorTrace Basic 2.1 software (all Unisense, Denmark). Measurements were performed at RT in stirred 2 ml—glass vials with a total reaction volume of 600 µl. Oxygen consumption was initiated by adding 30 nM (*bd*-I, *bo*_3_) or 120 nM (*bd*-II) of the respective enzyme to the equilibrated mixture containing 20 mM NaPi (pH 8.0), 50 mM NaCl, 0.02% DDM, 5 mM Dithiothreitol (DTT) and 200 µM Ubiquinone-1 (2,3-Dimethoxy-5-methyl-6-(3-methyl-2-buten-1-yl)-1,4-benzoquinone). A 10 min equilibration period was implemented prior to enzyme addition. Inhibition experiments were performed with 250 µM of the respective compound from 20 mM stock solution in DMSO before equilibration. Data analysis and visualization via Origin Lab Pro 9.5 (Additive GmbH, Germany) Data analysis included an unpaired, two-sided t-test of two samples to check that given residual activities were sufficiently different between proteins (at significance levels *p* < 0.05, 0.01 or 0.001). HQNO (2-heptyl-4-quinolinol 1-oxide) was purchased from biomol GmbH, Hamburg. Determination of apparent *K*_i_ (*K*_i_^app^) was performed under the same conditions but with a range of inhibitor concentrations from 250 µM to 0.2 nM to test protein/inhibitor ratio from 1/0.0067 up to 1/8333 for cytochromes *bd*-I and *bo*_3_ (tested at 30 nM). The *K*_i_^app^ value was adopted as the EC50 from the sigmoidal DoseRespFit. Further information can be found in Supporting Information Figures S2 to S5.

## Supplementary Information

Supplementary Information.
