# Supplementary material for: Short-chain aurachin D derivatives are selective inhibitors of E. coli cytochrome bd-I and bd-II oxidases
Source: Sci Rep. 2021 Dec 13;11:23852. doi: 10.1038/s41598-021-03288-7 (PMC8668966; doi:10.1038/s41598-021-03288-7)
Supplement: Supplementary file 1 — Supplementary Information. [file 41598_2021_3288_MOESM1_ESM.docx]

Supporting Information for

Short - chain aurachin D derivatives are selective inhibitors of *E. coli* cytochrome *bd*‑I and *bd*-II oxidases

Contents

[1. Compound screening results 2](#_Toc80097966)

[2. Dose response curves: K_i_^app^ determination 3](#_Toc80097968)

[3. Synthesis 7](#_Toc80097969)

## Compound screening results

### Inhibitory activity at 250µM


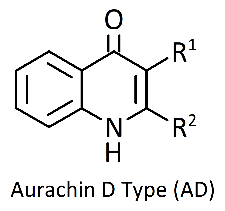

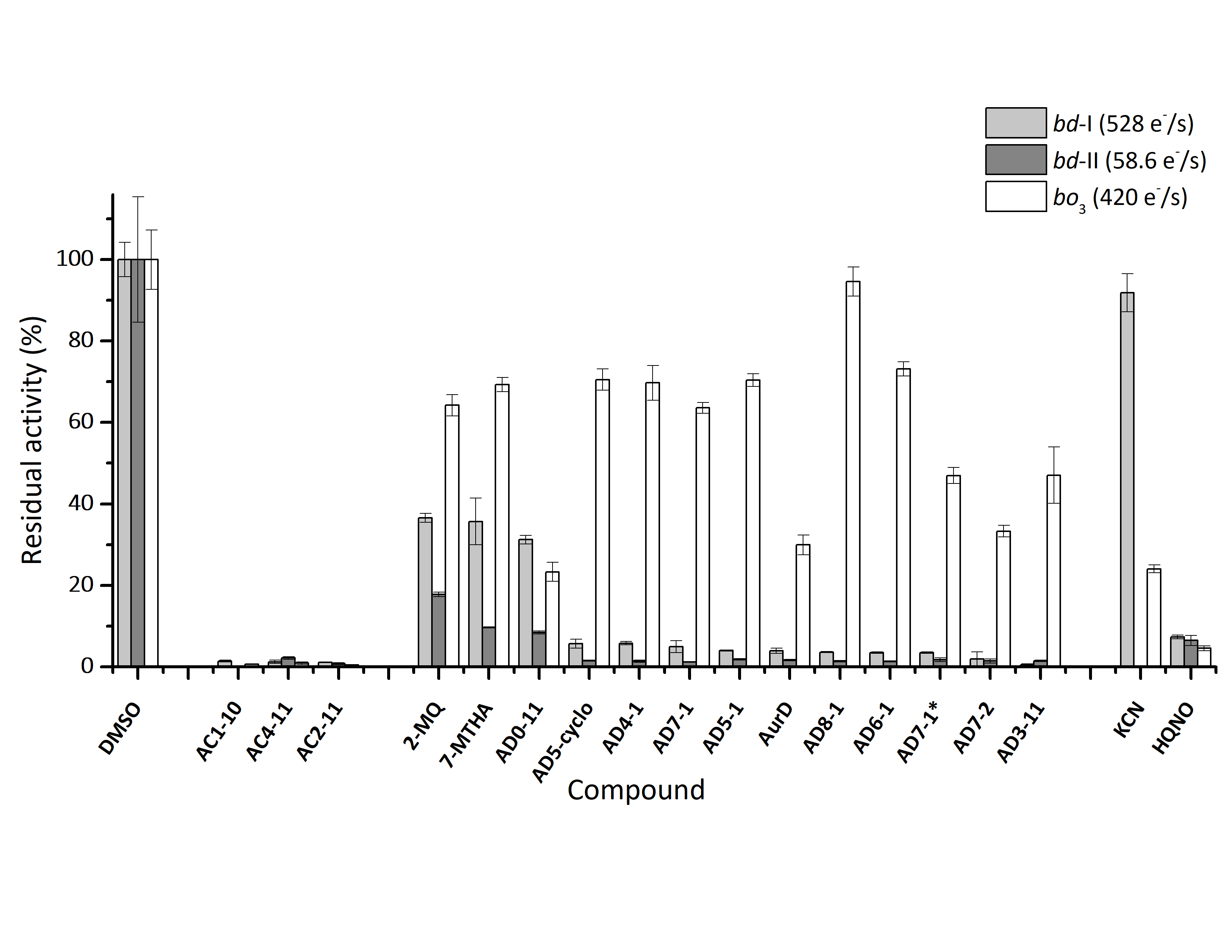

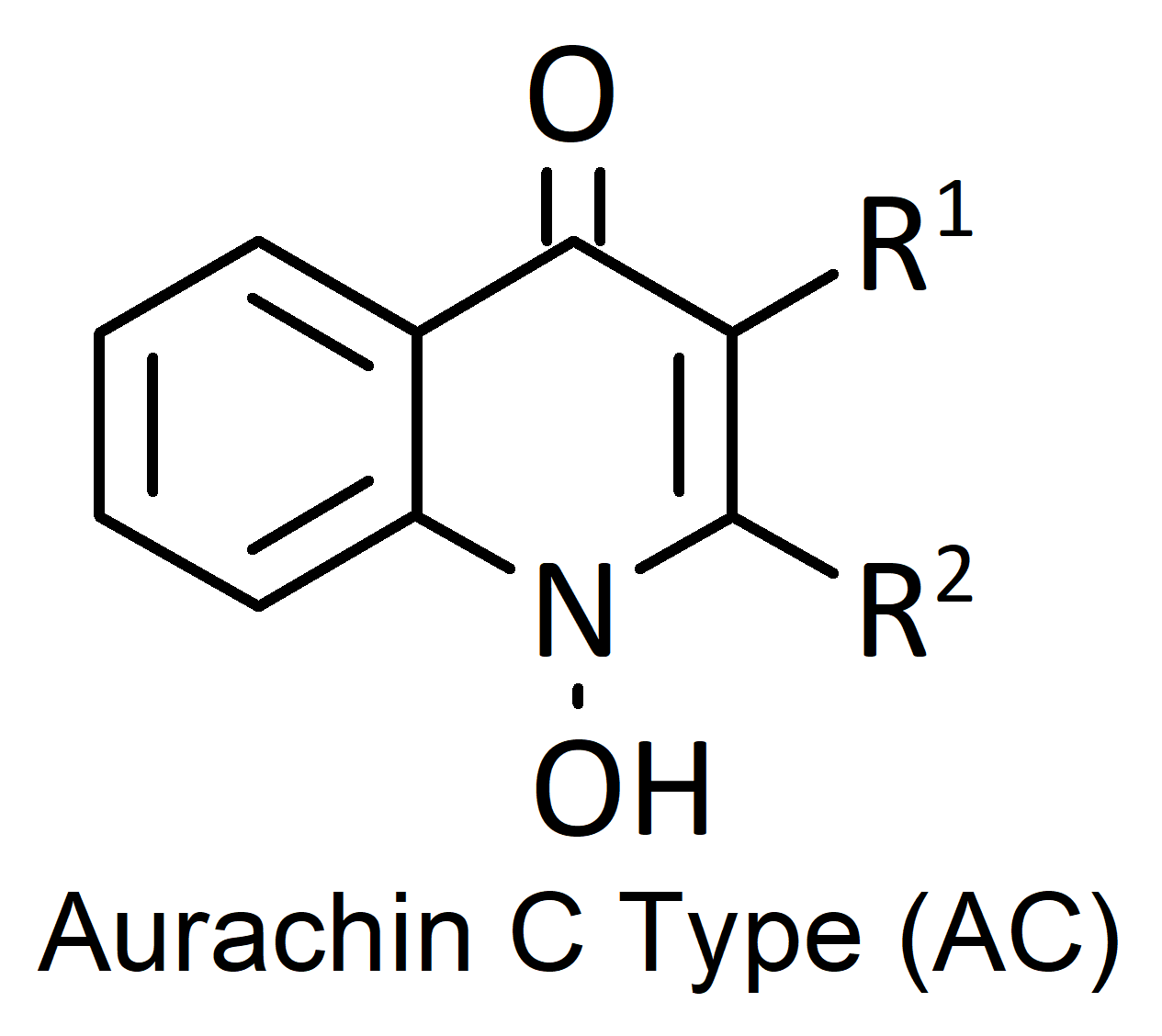


**Aurachin D Type (AurD)**

**Aurachin C Type (AurC)**

**Figure S-1.** Screening results for *bd*-I (light-grey), *bd*-II (dark-grey) and *bo*_3_ (white) with decreasing residual activity for *bd*-I from left to right. Inhibition assay using test compounds at 250µM in presence of 200µM ubiquinone-1 and 5mM dithiothreitol. Oxygen reduction activity was calculated from oxygen consumption rates at 30nM (*bd*-I, *bo*_3_) or 120nM (*bd*-II) oxidase at RT for each experiment. 100% activity = adding corresponding volume of DMSO. KCN at 20µM (*bd*-I, *bo*_3_), was used as a *bo*_3_ selective inhibitor. HQNO at 250µM represents an active, non-selective inhibitor for all three oxidases. Presented data are mean ± S.E.M. (n=3) For detailed data, please refer to Supplement Information Table SI-1.

**Table S-1.** Bioactivity of AurC-type and AurD-type compounds at 250µM. Residual quinol oxygen reduction activity for *E. coli* cytochrome *bd*-I*, bd*-II and *bo*_3_ oxidase. Inhibition assay using test compounds at 250µM in presence of 200µM ubiquinone-1 and 5mM dithiothreitol. Oxygen reduction activity was calculated from oxygen consumption rates at 30nM (*bd*-I, *bo*_3_) or 120nM (*bd*-II) at RT for each experiment. 100% activity = adding corresponding volume of DMSO. KCN at 20µM (*bd*-I, *bo*_3_) was used as a cytochrome *bo*_3_ selective inhibitor (*10mM for *bd*-II). HQNO at 250µM represents an active, non-selective inhibitor for all three oxidoreductases. Presented data are mean ± S.E.M. (n=3)

| **Compound** | **Rest activity in % of control activity** | | |
| --- | --- | --- | --- |
|  | ***bd*-I** | ***bd*‑II** | ***bo*_3_** |
| **DMSO** | 100 ± 4.2 | 100 ± 15.4 | 100 ± 7.3 |
| **AC1-10** | 1.39 ± 0.19 | n.a. | 0.626 ± 0.08 |
| **AC4-11** | 1.19 ± 0.42 | 2.14 ± 0.35 | 0.863 ± 0.29 |
| **AC2-11** | 1.09 ±0.09 | 0.73 ± 0.21 | 0.445 ± 0.08 |
| **2-MQ** | 36.6 ±1.1 | 17.8 ± 0.55 | 64.2 ± 2.6 |
| **7-MTHA** | 35.7 ± 5.7 | 9.6 ± 0.16 | 69.3 ± 1.7 |
| **AD5-cyclo** | 5.67 ± 1.14 | 1.51 ± 0.15 | 70.5 ± 2.6 |
| **AD0-11** | 31.2 ±1.1 | 8.4 ± 0.36 | 23.3 ± 2.3 |
| **AD4-1** | 5.80 ± 0.39 | 1.33 ± 0.26 | 69.7 ± 4.3 |
| **AD7-1** | 4.90 ± 1.5 | 1.18 ± 0.12 | 63.6 ± 1.3 |
| **AD5-1** | 4.00 ± 0.13 | 1.85 ± 0.18 | 70.4 ± 1.5 |
| **AD8-1** | 3.59 ± 0.11 | 1.36 ± 0.21 | 94.6 ± 3.6 |
| **AD6-1** | 3.45 ± 0.19 | 1.30 ± 0.17 | 73.2 ± 1.7 |
| **AD7-1*** | 3.46 ± 0.17 | 1.71 ± 0.48 | 47.0 ± 1.9 |
| **AD7-2** | 1.89 ± 1.8 | 1.40 ± 0.49 | 33.3 ± 1.4 |
| **AurD** | 3.93 ± 0.64 | 1.62 ± 0.19 | 29.9 ± 2.4 |
| **AD3-11** | 0.453 ± 0.28 | 1.45 ± 0.18 | 47.1 ± 7.0 |
| **KCN** | 91.8 ± 4.7 | 12.2 ± 1.5 * | 24.03 ± 0.98 |
| **HQNO** | 7.29 ± 0.45 | 6.44 ± 1.3 | 4.53 ± 0.58 |

## Dose response curves: K_i_^app^ determination

| Parameter initialization | | |
| --- | --- | --- |
| A1 | = bottom asymptote | : fixed at 0 |
| A2 | = top asymptote | : fixed at 1 |
| LOG_x_0 | = center | : automated parameter initialization |
| p | = hill slope | : automated parameter initialization |
| Derived parameters | |  |
| EC50 | = 10^Log_x_0 | : K_i_^app^ in µM |

Dose response curves were created to rank the compounds due to their inhibition potency. Inhibitors were titrated in presence of 30nM *bd*-I. From Clark-type electrode monitored oxygen consumption, resulting oxygen reduction activity with 200µM Q1, reduced with 2mM DTT, was calculated as turnover in e^-^/s. Turnover values were normalized, followed by a curve fitting with DoseResp Fit (1) in Origin LabPro9.5 (Additive GmbH, Germany). Data points are mean of minimum three independent measurements ± S.E.M.

1. $y=A1+\frac{A1-A2}{1+{10}^{({Log}_{x}0-x)p}}$


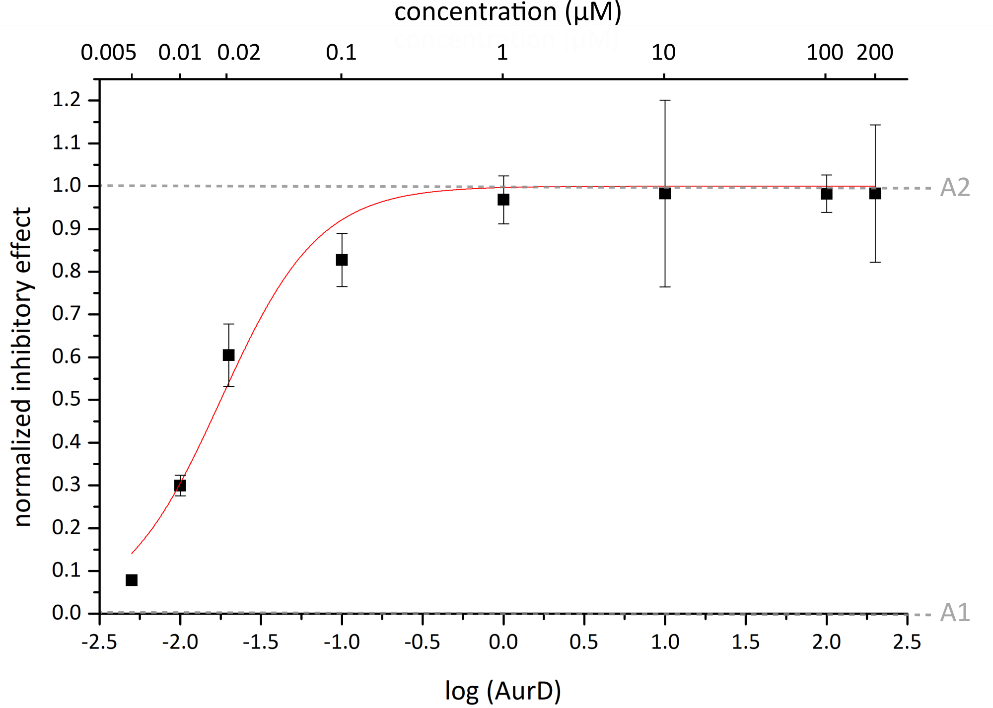


AurD K_i_^app^ 0.018 ± 0.002µM

**Figure S-2:** K_i_^app^ determination for AurD and *E. coli bd*-I*.* Half-logarithmic plotting of normalized inhibitory effect on *bd*-I over AurD concentration (1nM - 200µM), followed by sigmoidal fit (red line) with DoseResp fit (Origin LabPro9.5). Each point represents mean ± S.E.M. (n=3).


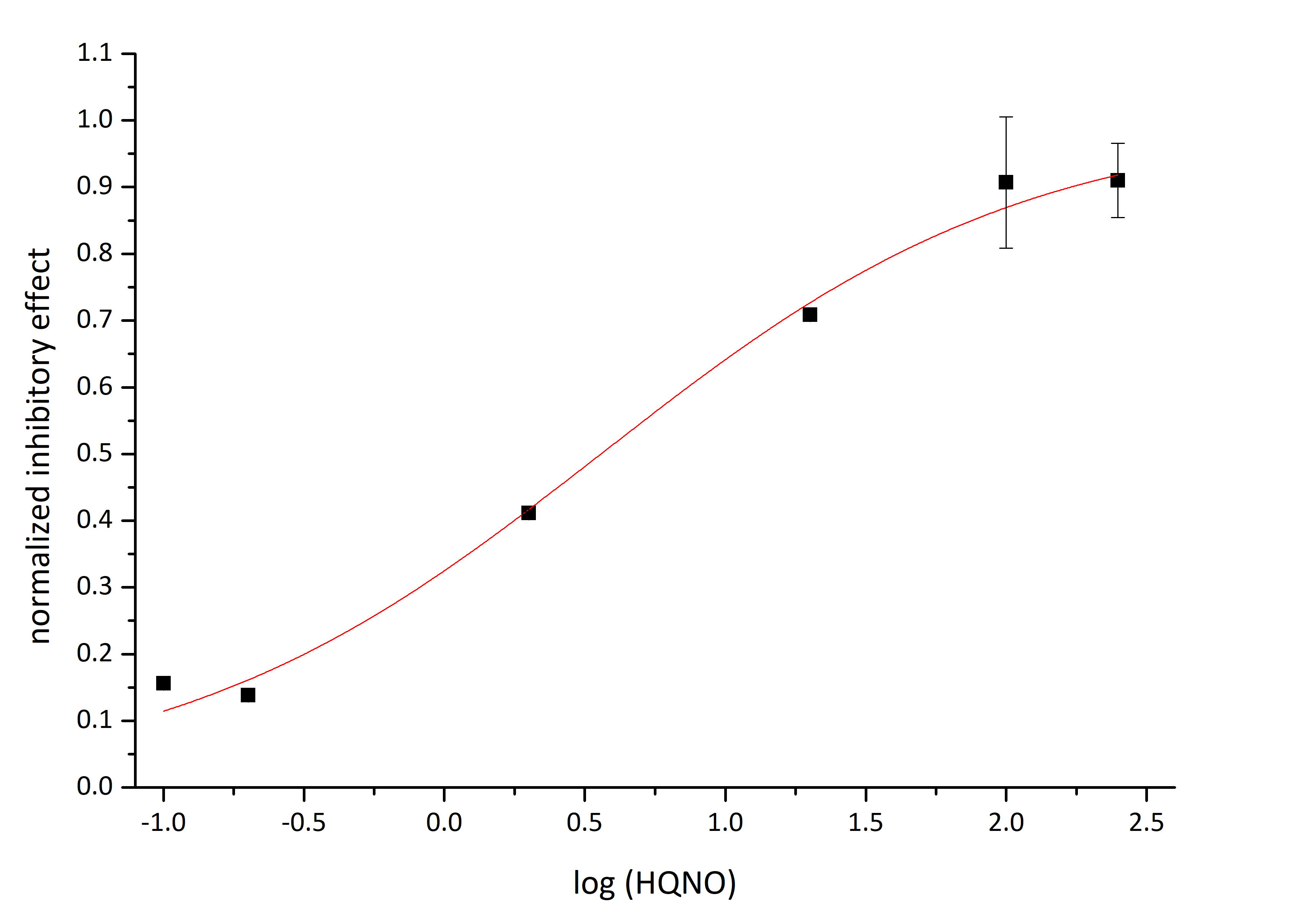

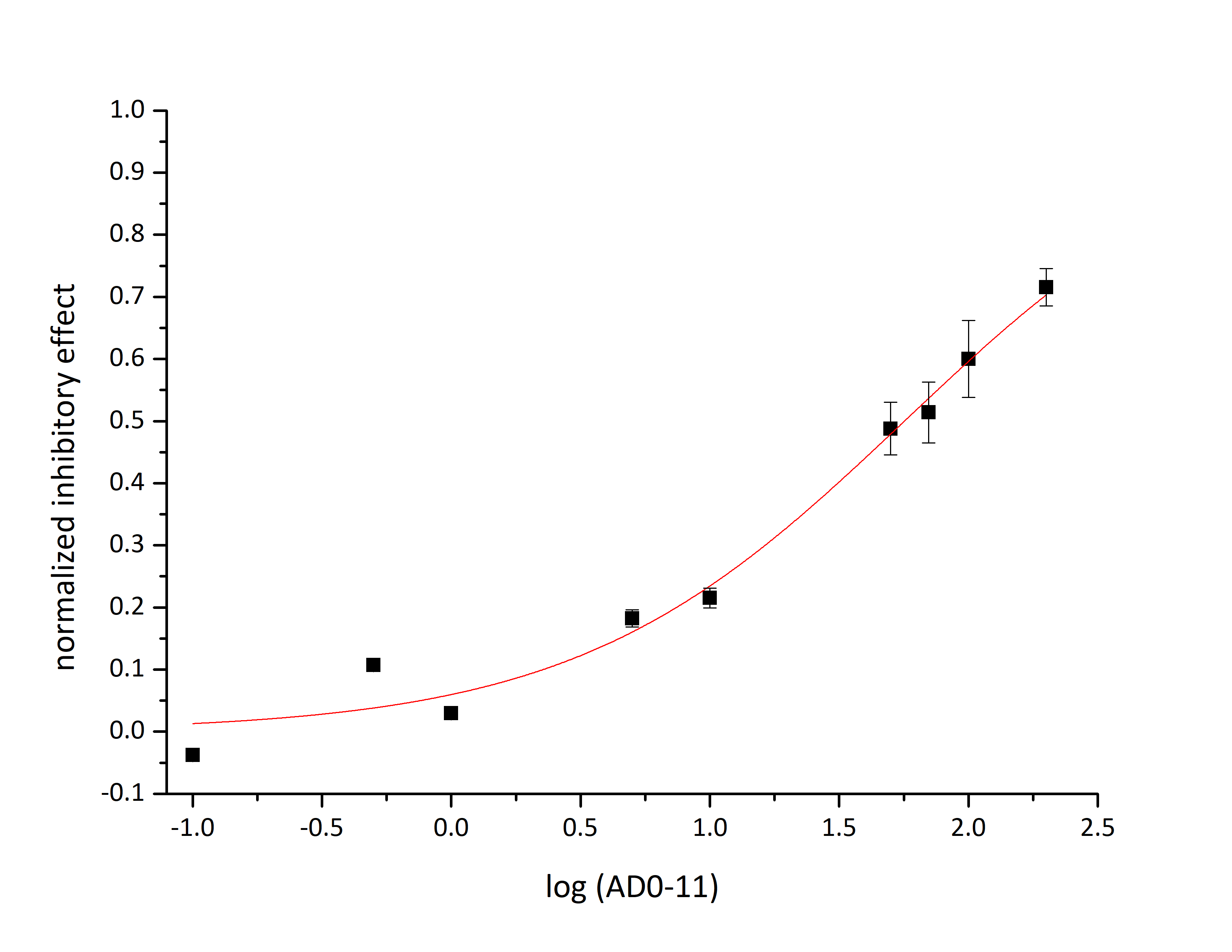


HQNO K_i_^app^ 3.6 ± 0.5µM

AD0-11 K_i_^app^ 56.6 ± 5.9µM


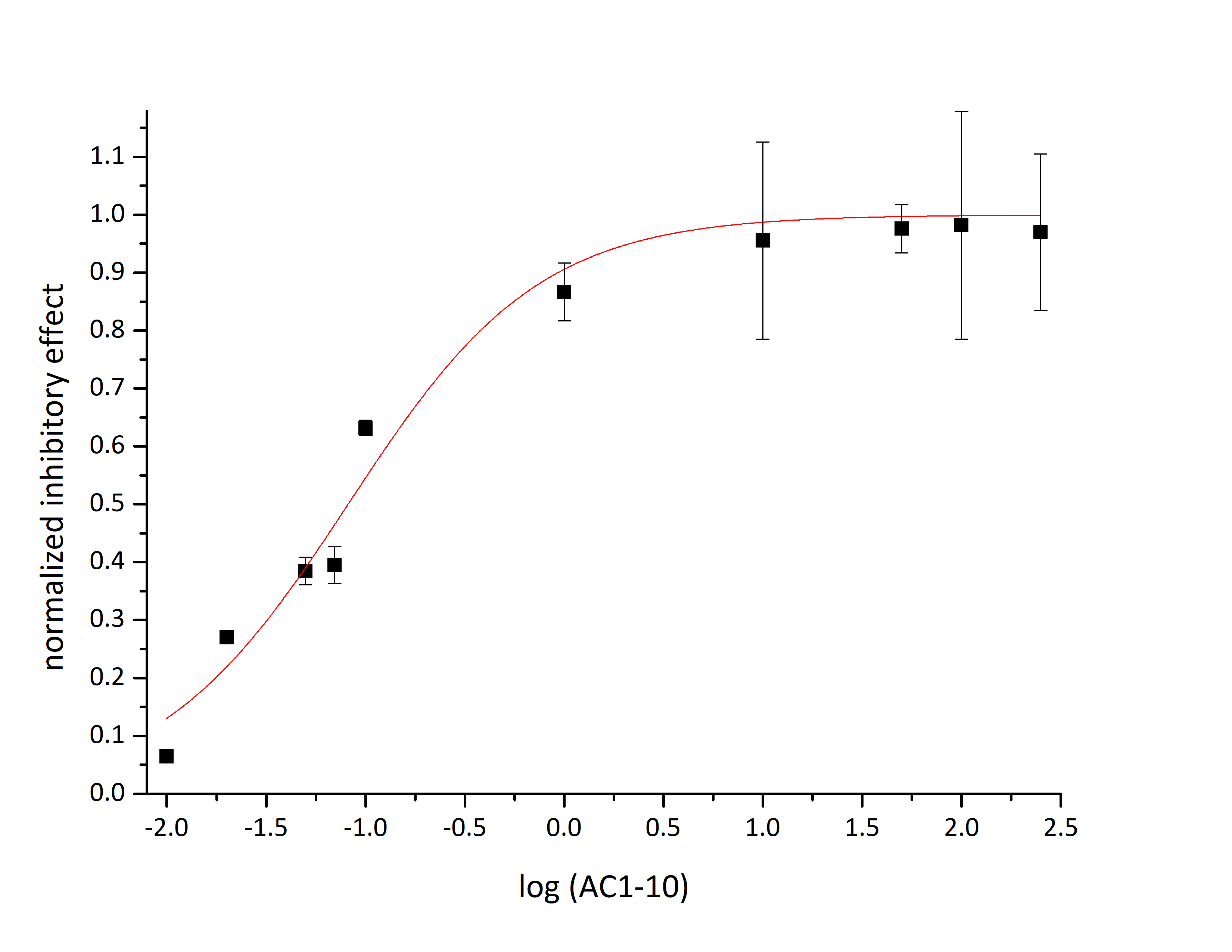

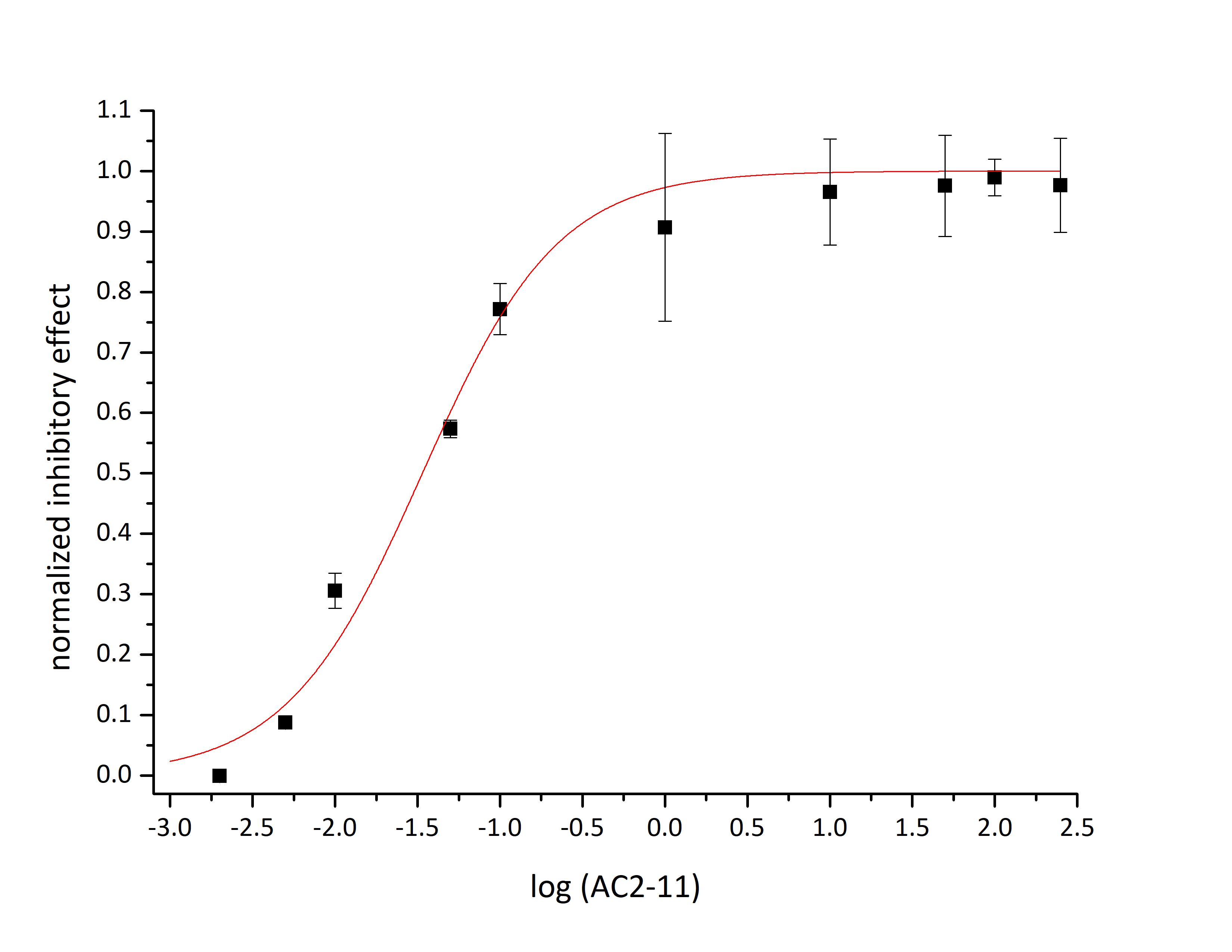


AC2-11 K_i_^app^ 0.034 ± 0.006µM

AC1-10 K_i_^app^ 0.082 ± 0.011µM


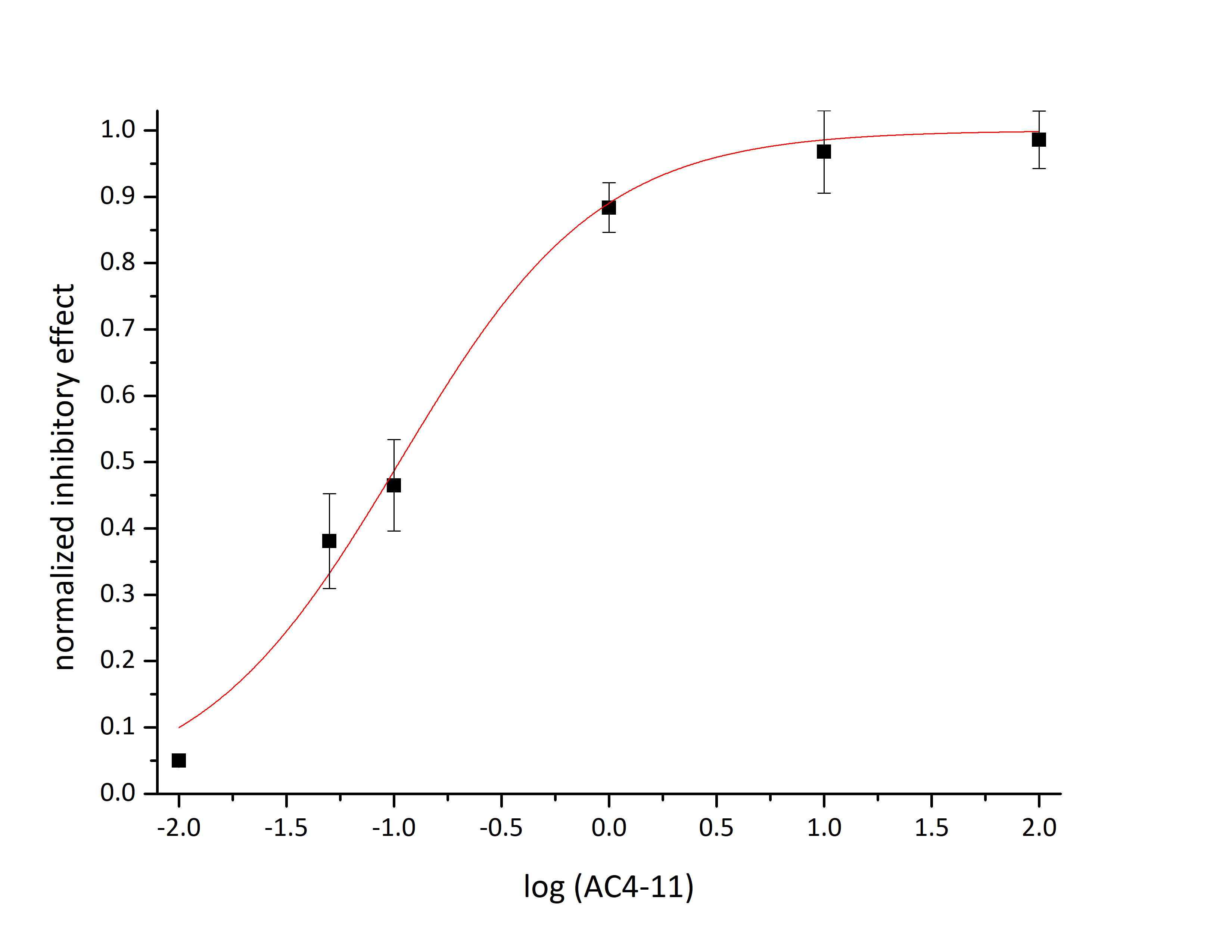

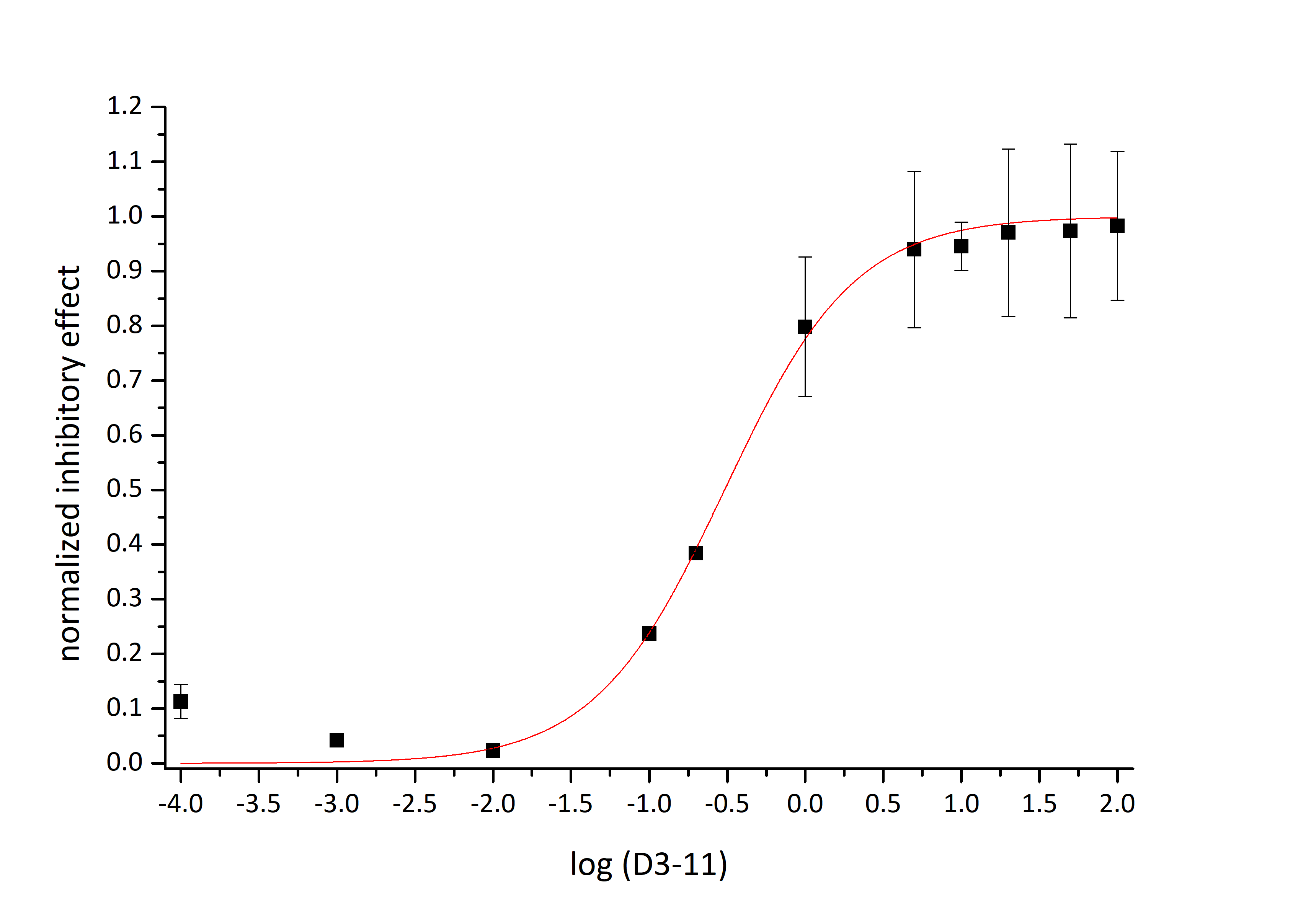

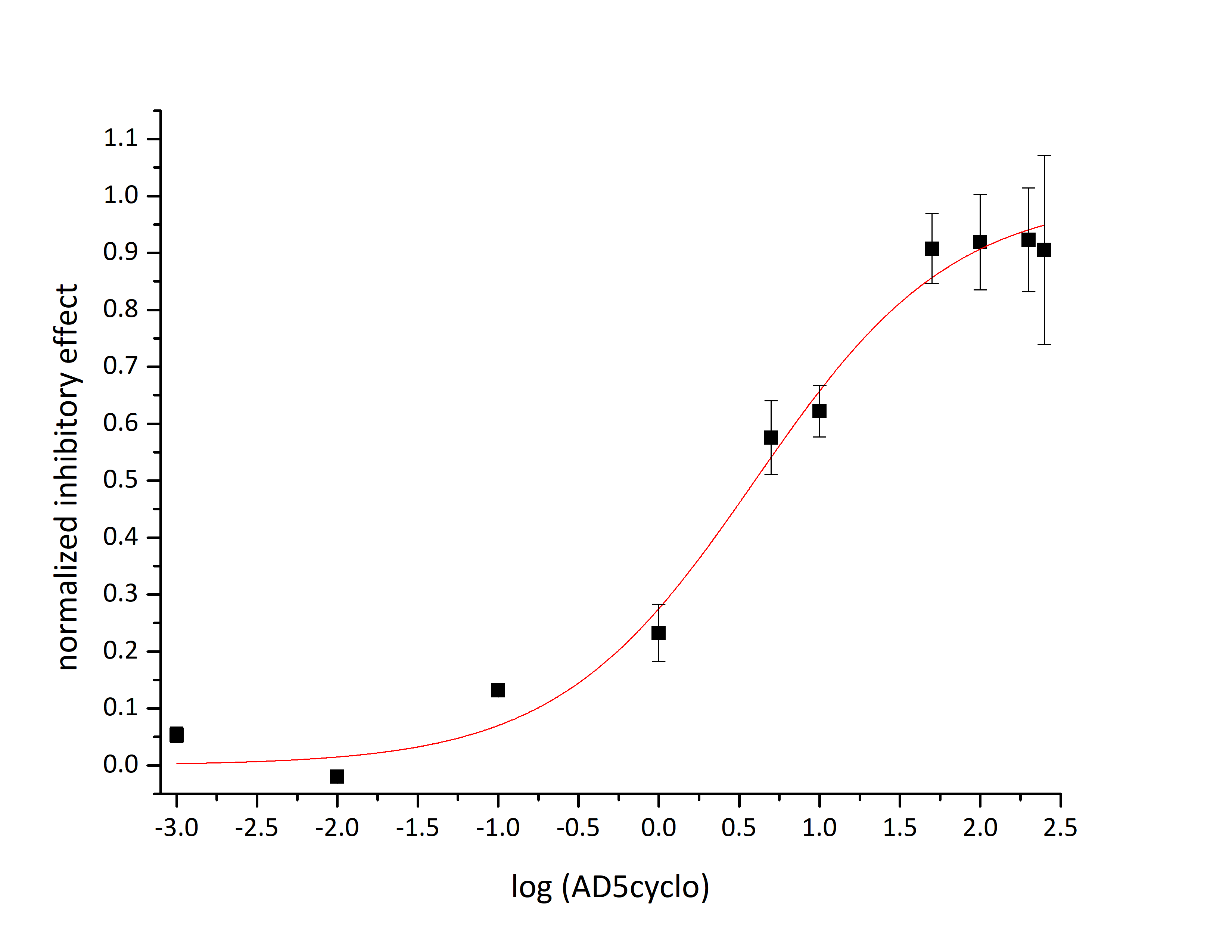

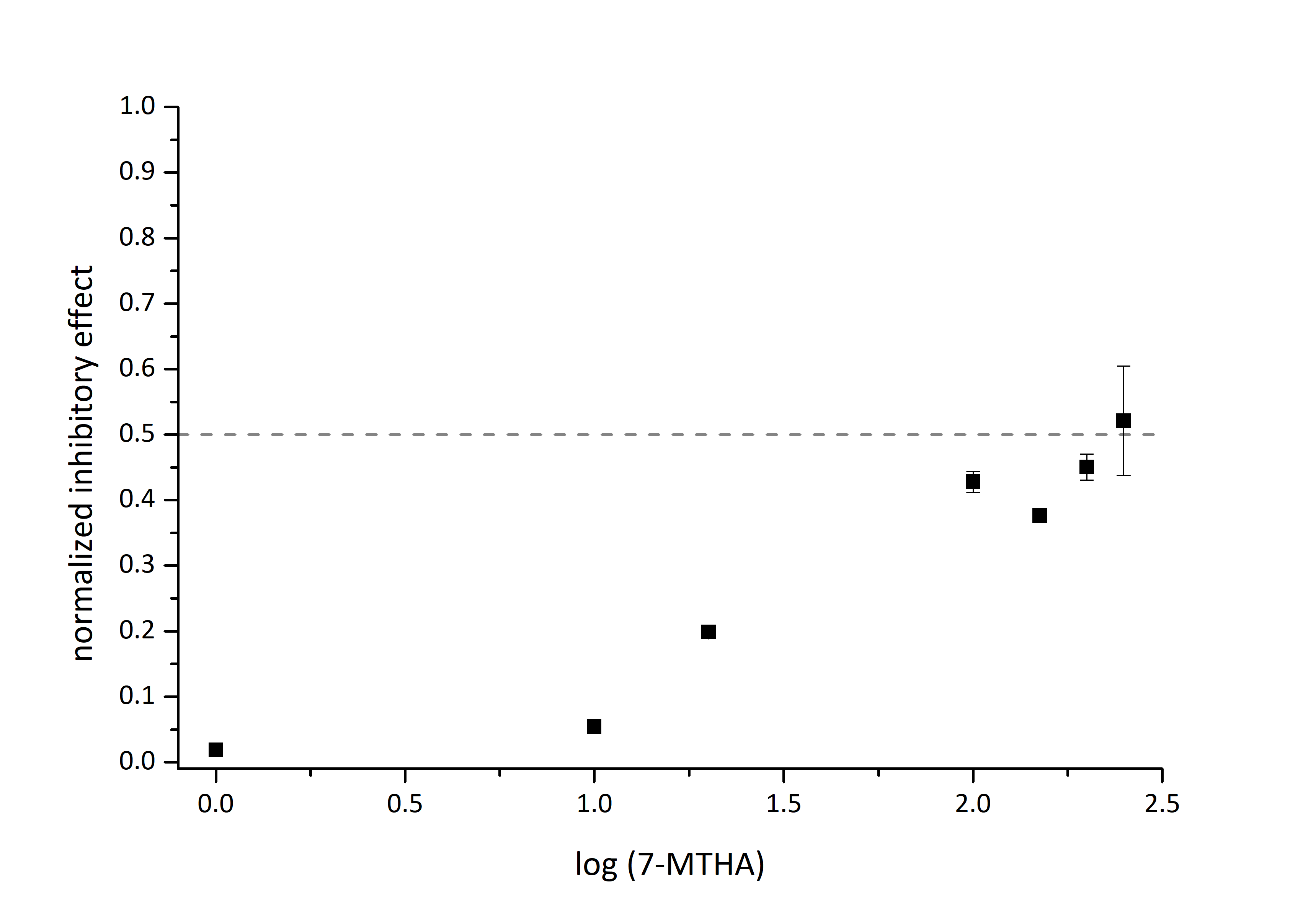

**Figure S-3**: Half- logarithmic dose response curves for *E. coli* cytochrome *bd*-I oxidase. K_i_^app^ determined by functional oxygen reductase assay as for AurD. 30nM *bd*-I in presence up to 250µM inhibitor. Each point represents mean ± S.E.M. (n=3). Sigmoidal fit (red line) with DoseResp fit (Origin LabPro9.5).

*y = 0.5*

7-MTHA K_i_^app^ > 250µM

AD5cyclo K_i_^app^ 4.0 ± 0.6µM

AD3-11 K_i_^app^ 0.30 ± 0.04µM

AC4-11 K_i_^app^ 0.106 ± 0.013µM

**
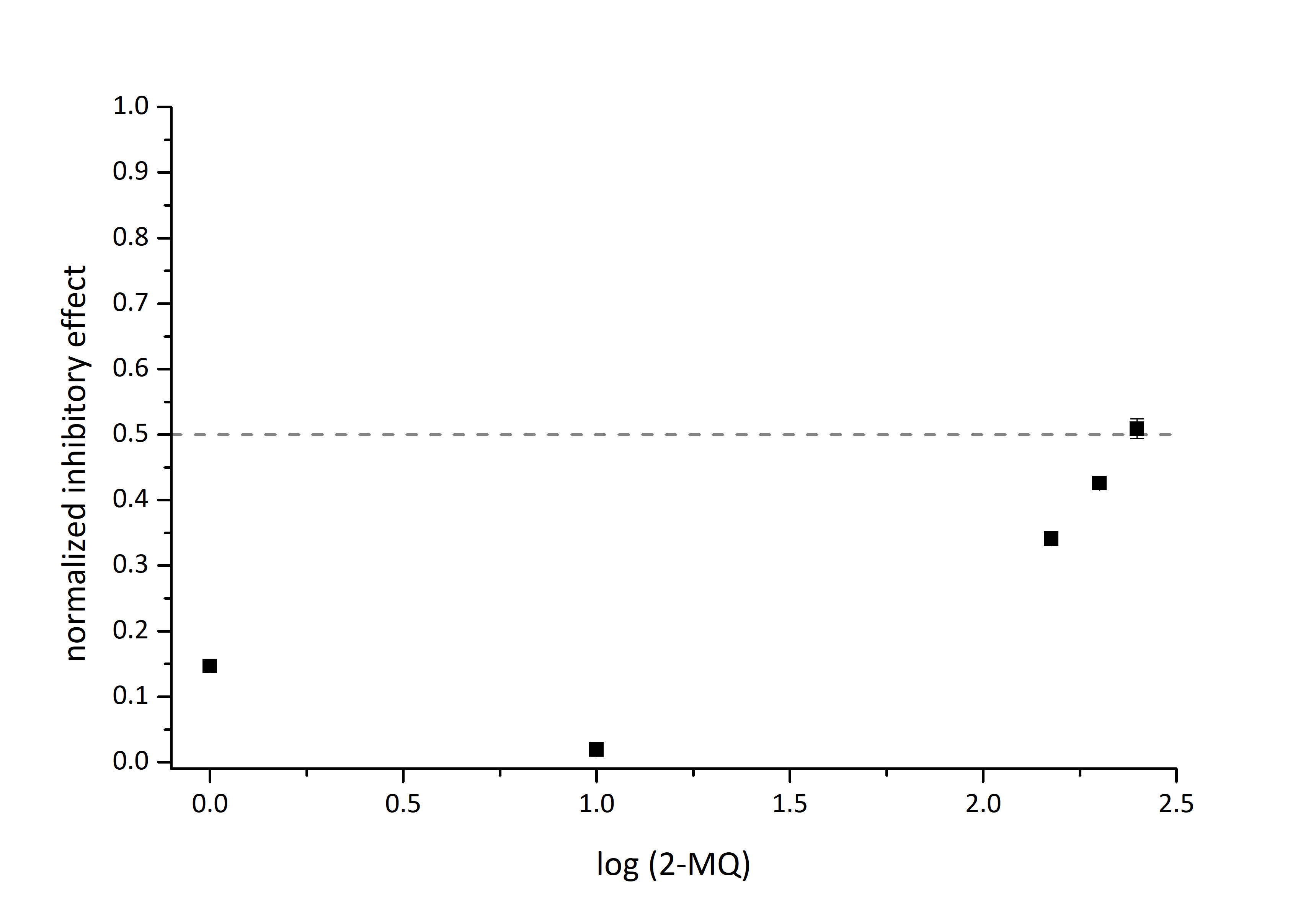

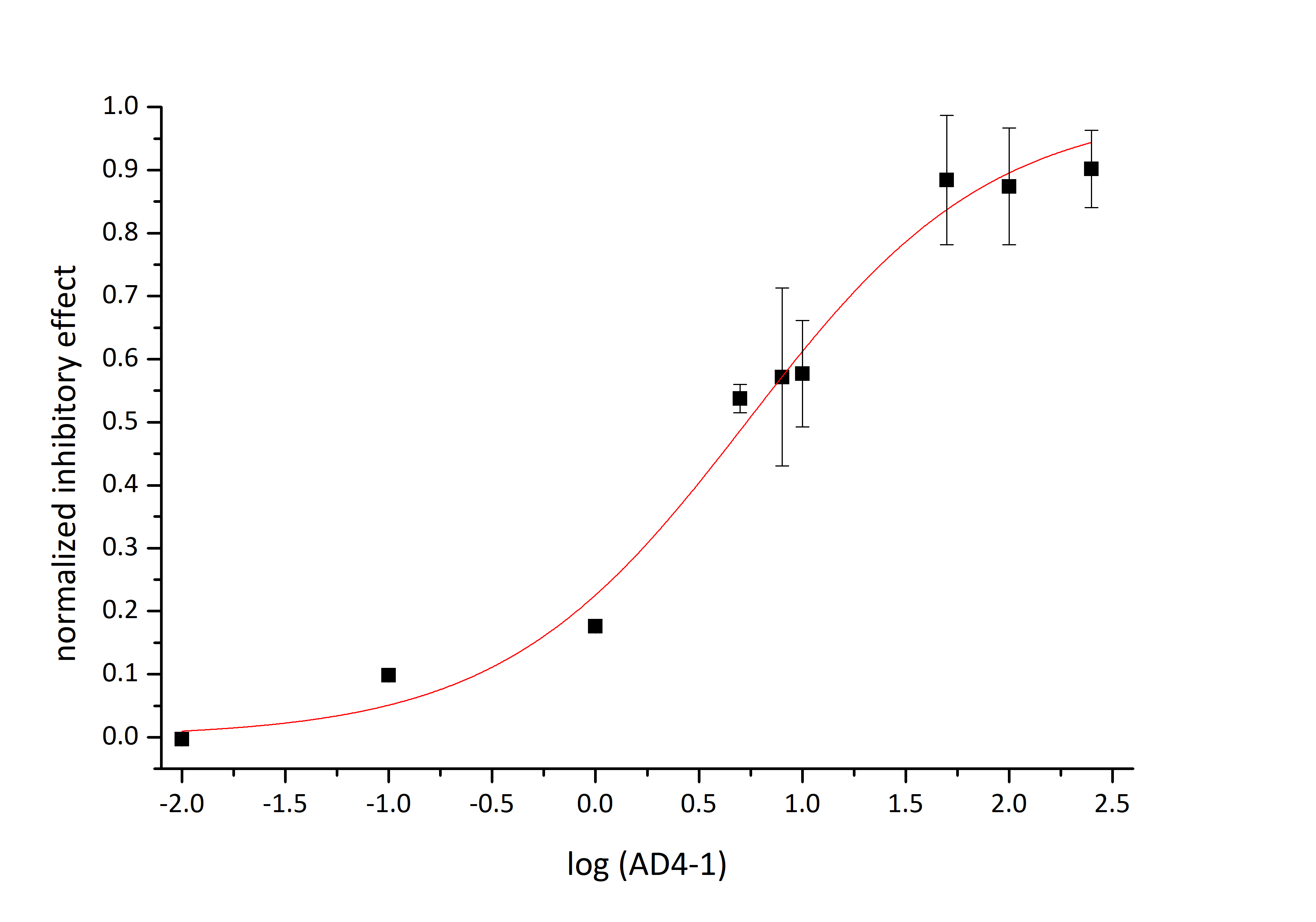
**
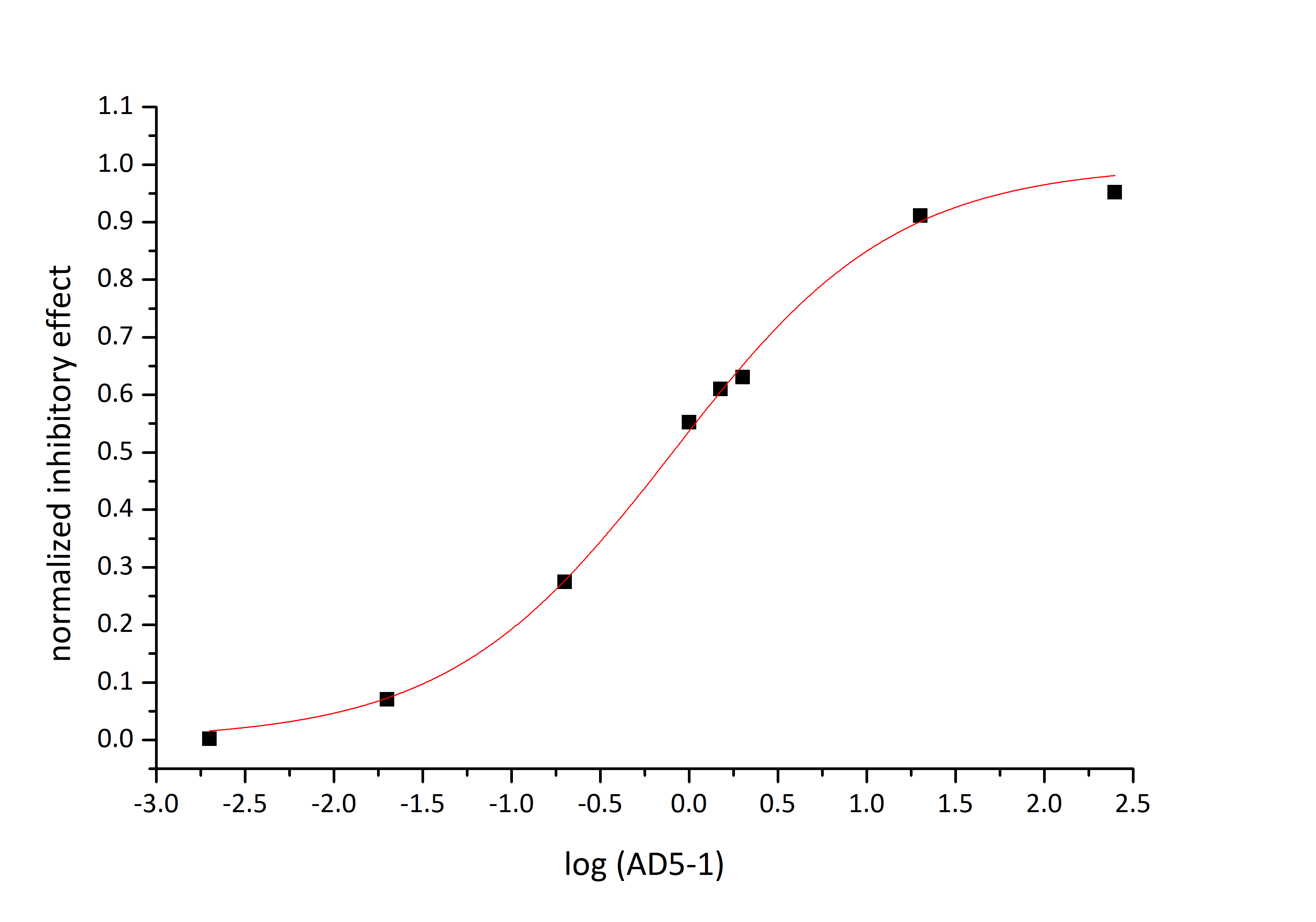

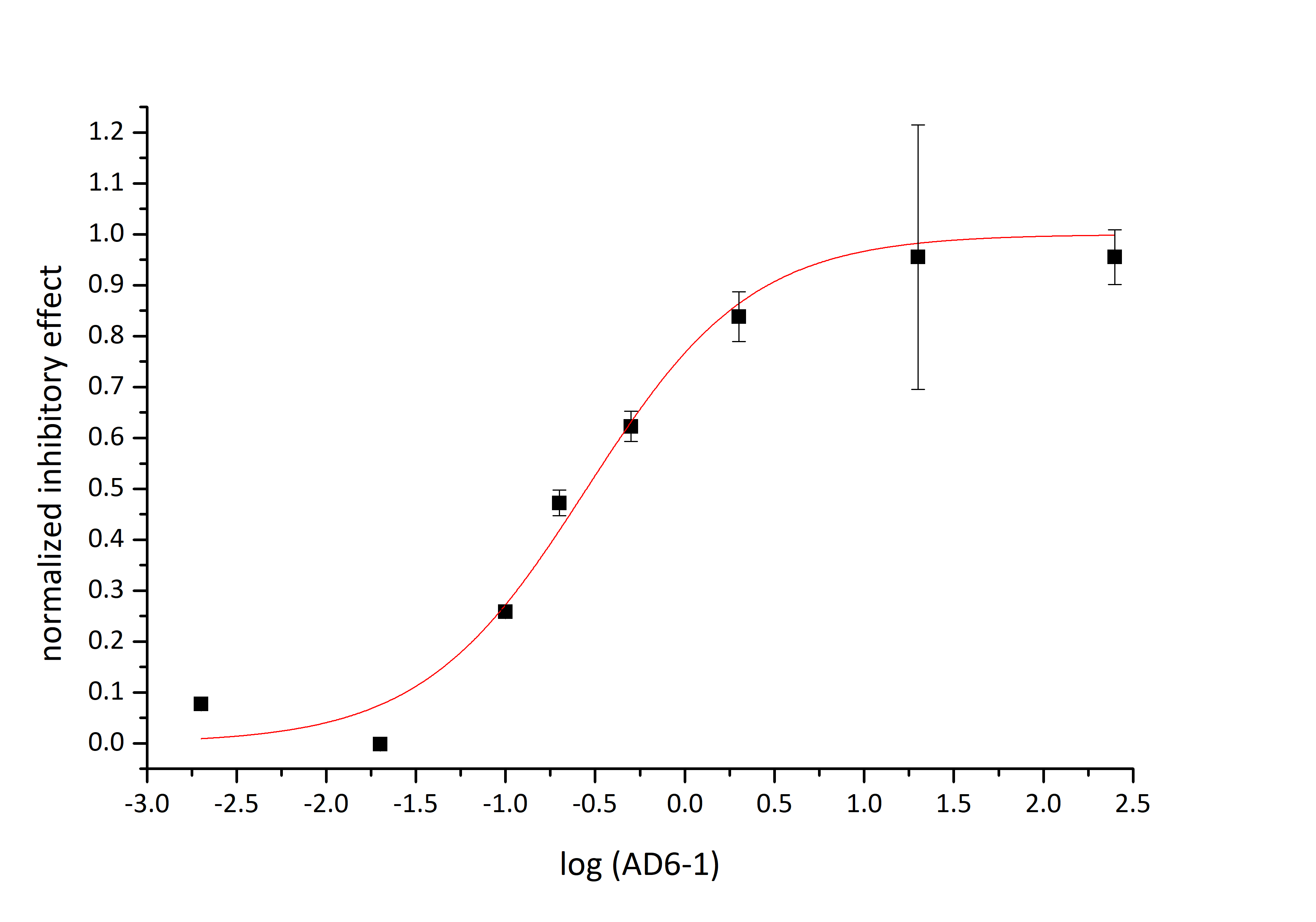


*y = 0.5*

AD4-1 K_i_^app^ 5.4 ± 0.7µM

2-MQ K_i_^app^ > 250µM

AD6-1 K_i_^app^ 0.28 ± 0.04µM

AD5-1 K_i_^app^ 0.81 ± 0.04µM


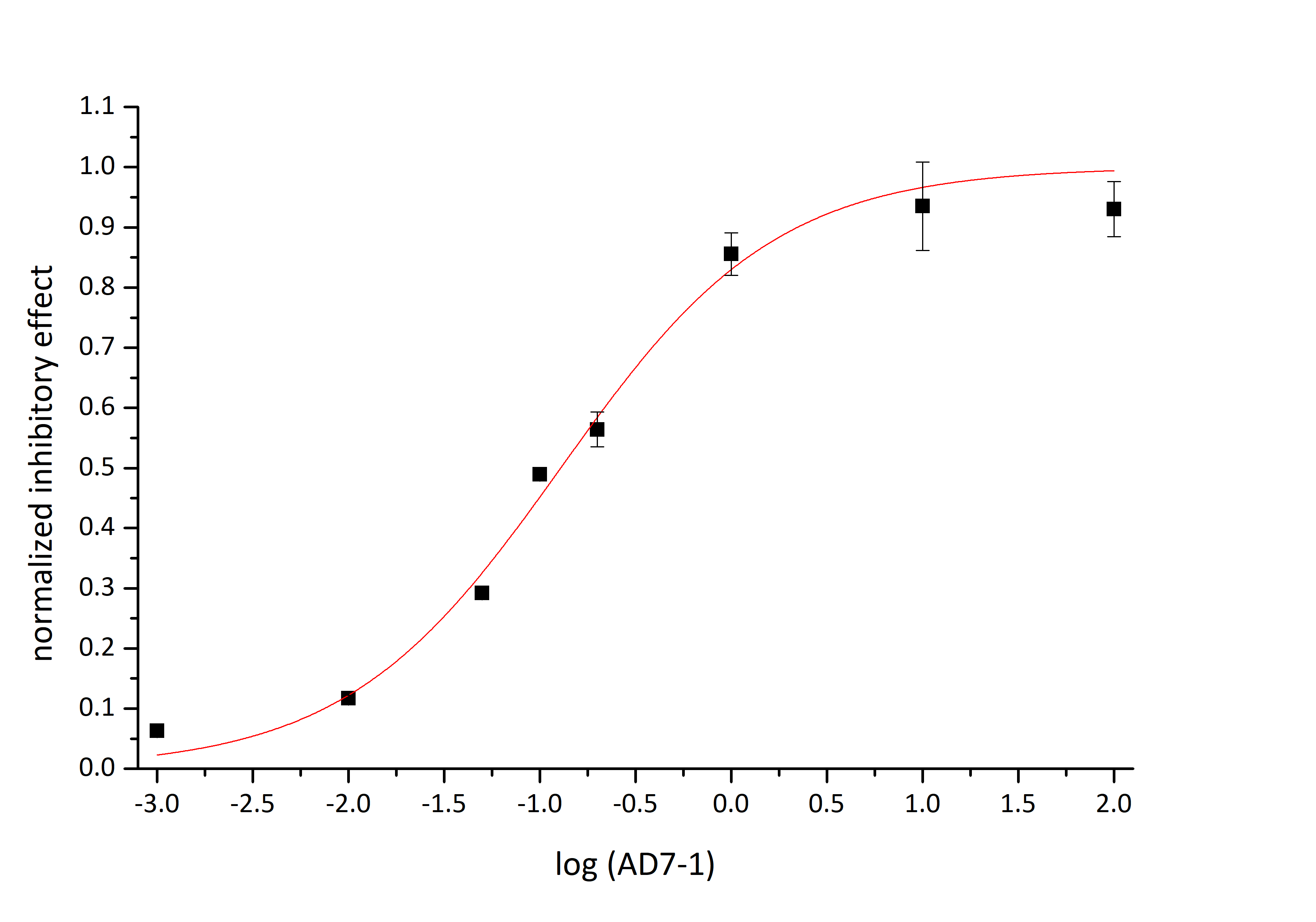

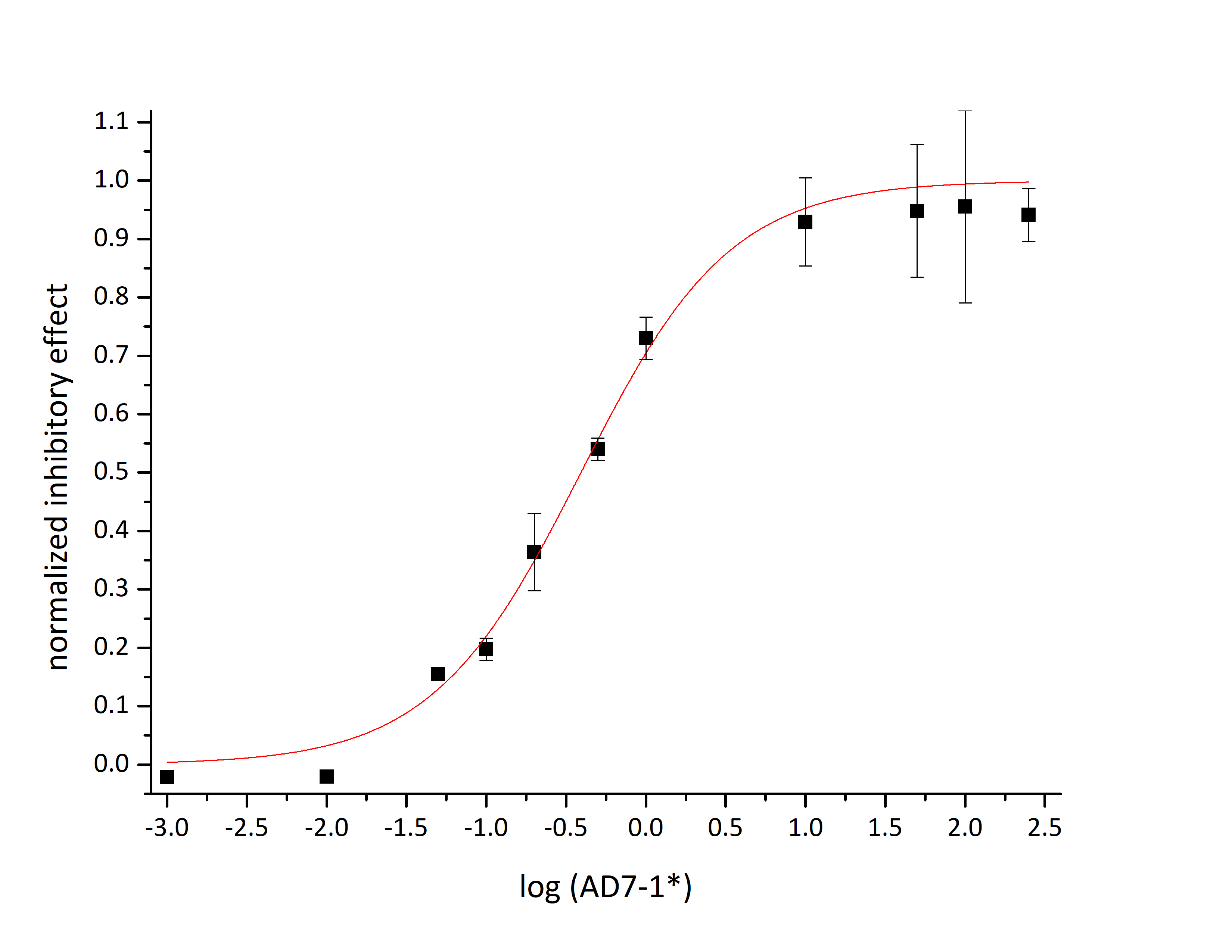


AD7-1* K_i_^app^ 0.39 ± 0.04 µM

AD7-1 K_i_^app^ 0.13 ± 0.02µM


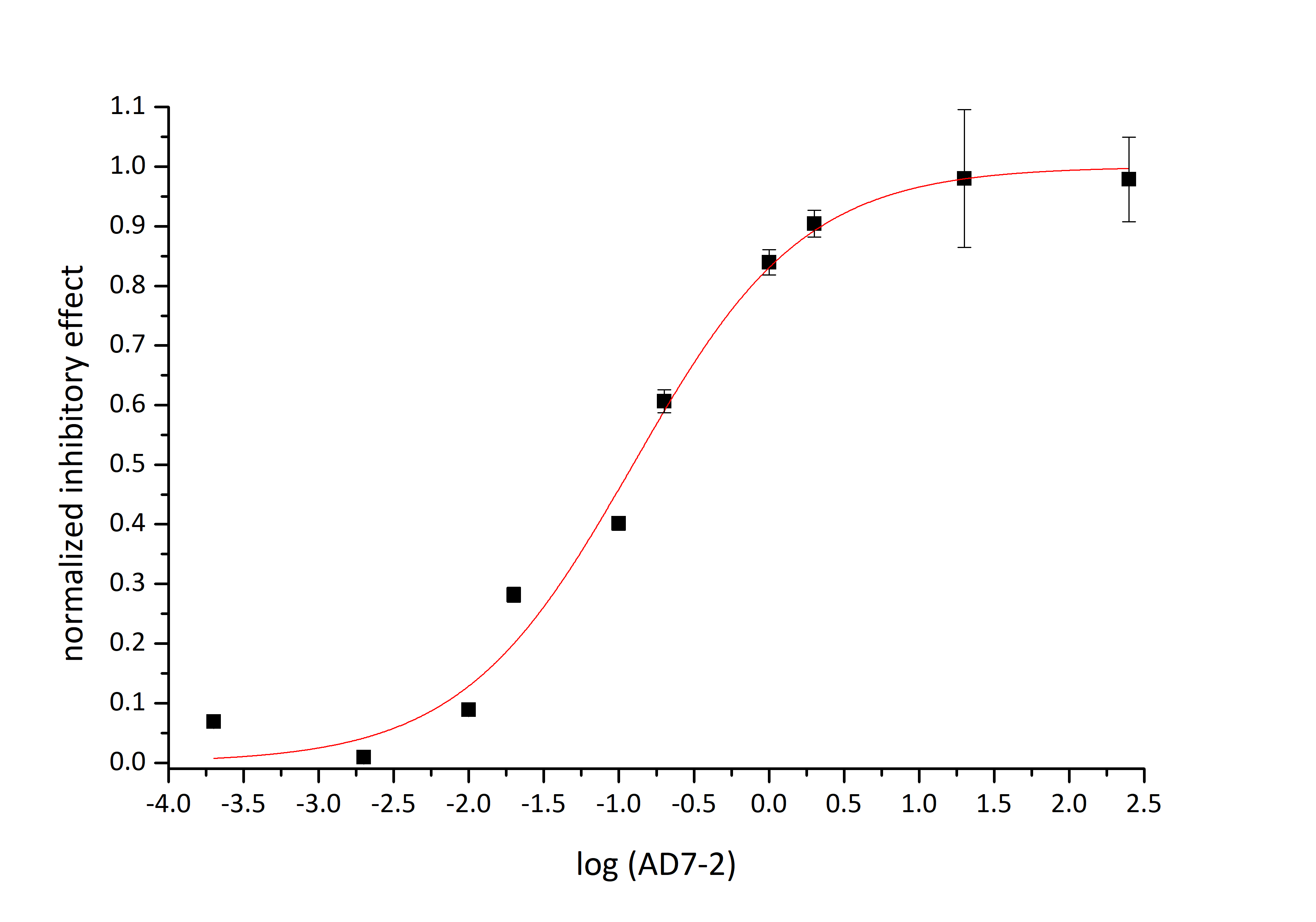

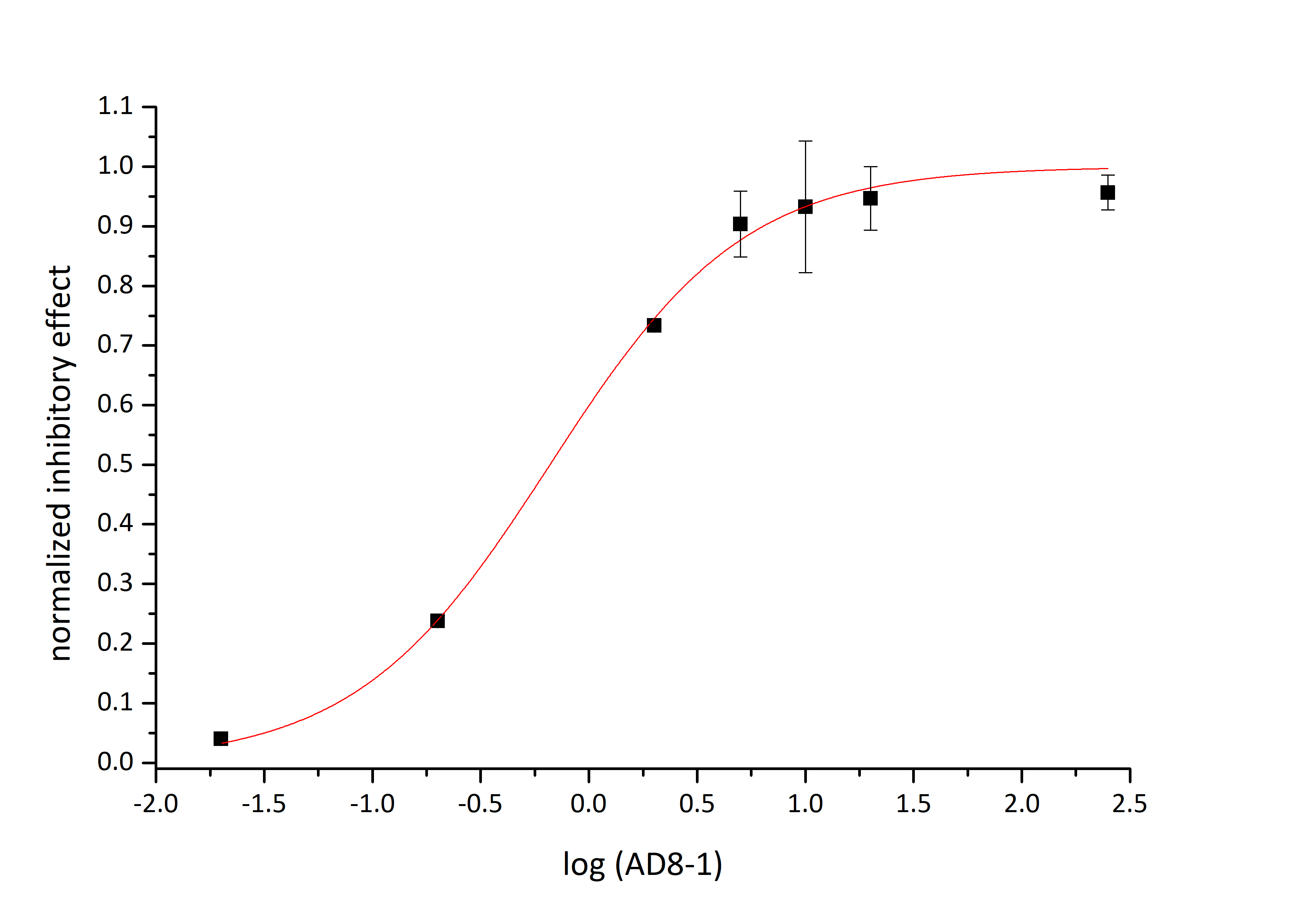


AD7-2 K_i_^app^ 0.12 ± 0.02µM

AD8-1 K_i_^app^ 0.66 ± 0.06µM

**Figure S-4**: Half- logarithmic dose response curves for *E. coli* cytochrome *bd*-I oxidase. K_i_^app^ determined by functional oxygen reductase assay as for AurD. 30nM *bd*-I in presence up to 250µM inhibitor. Each point represents mean ± S.E.M. (n=3). Sigmoidal fit (red line) with DoseResp fit (Origin LabPro9.5).


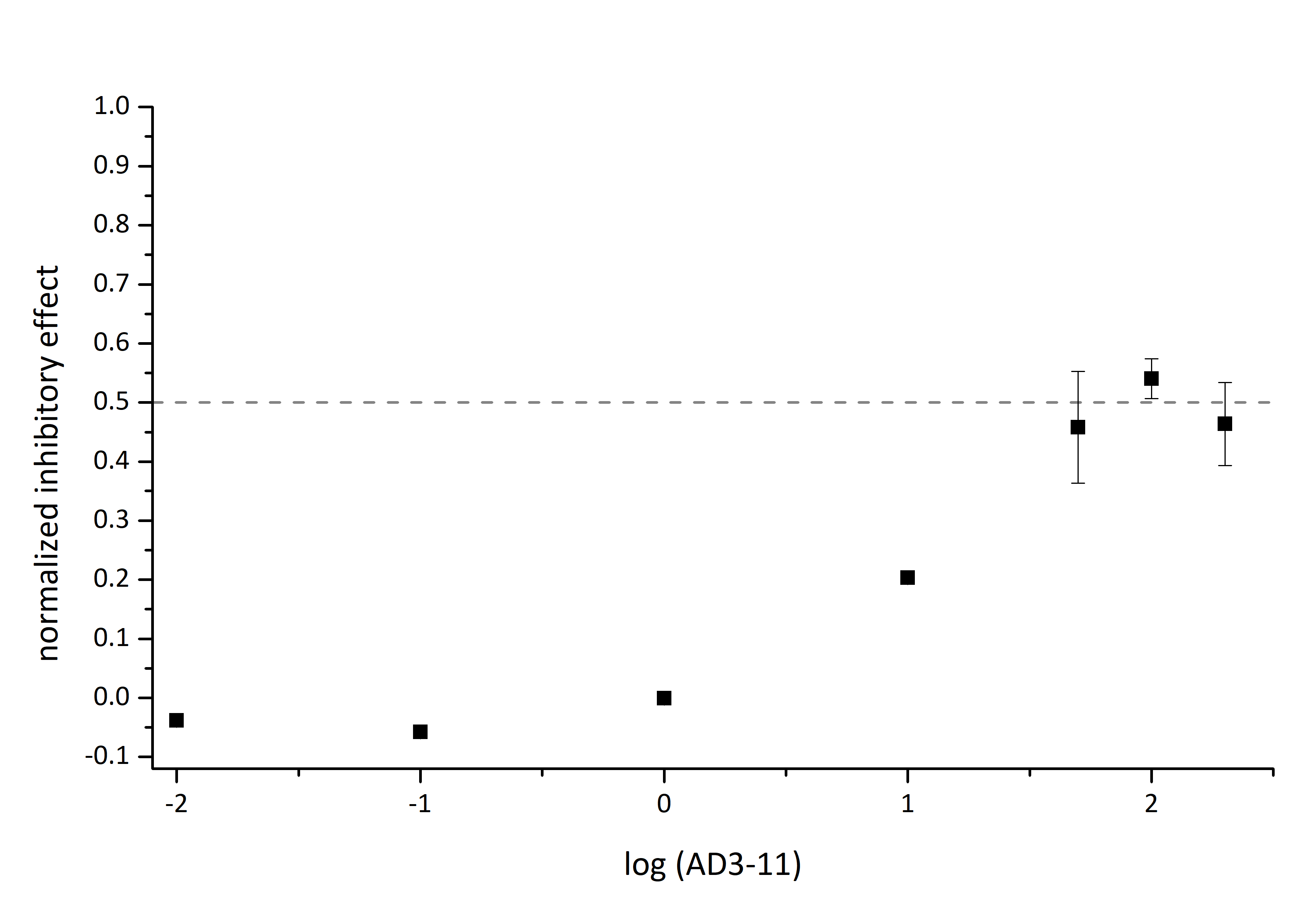

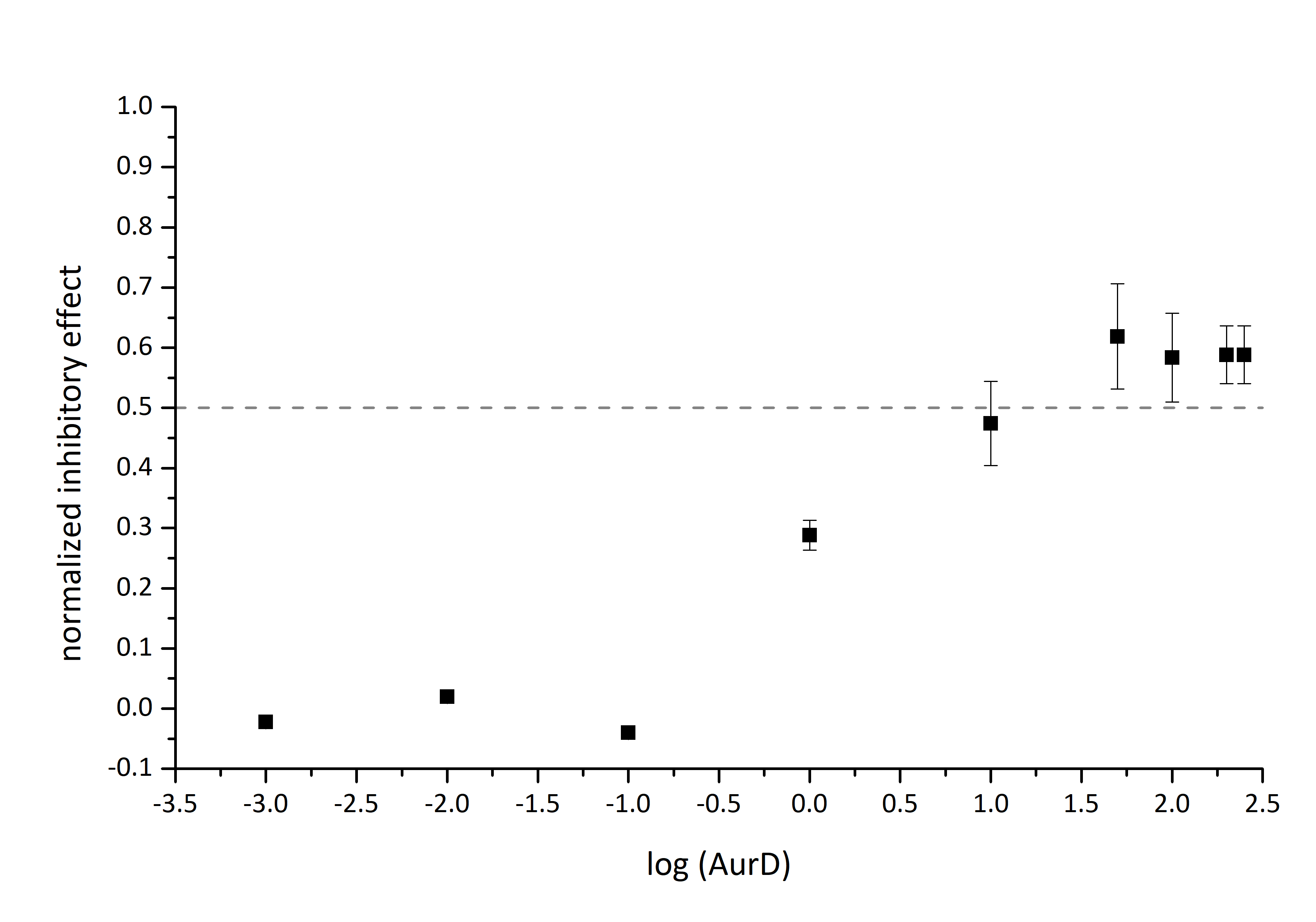

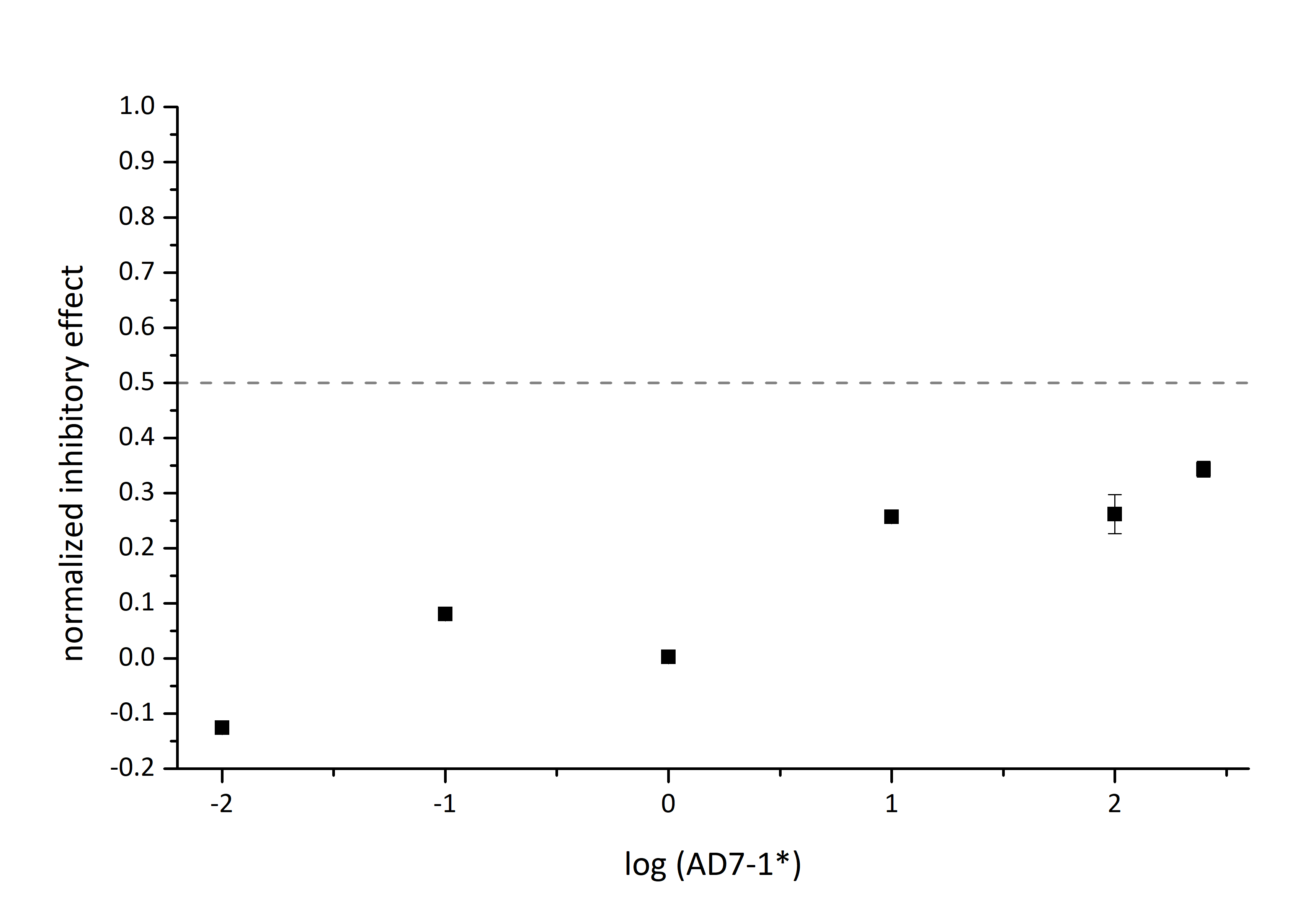

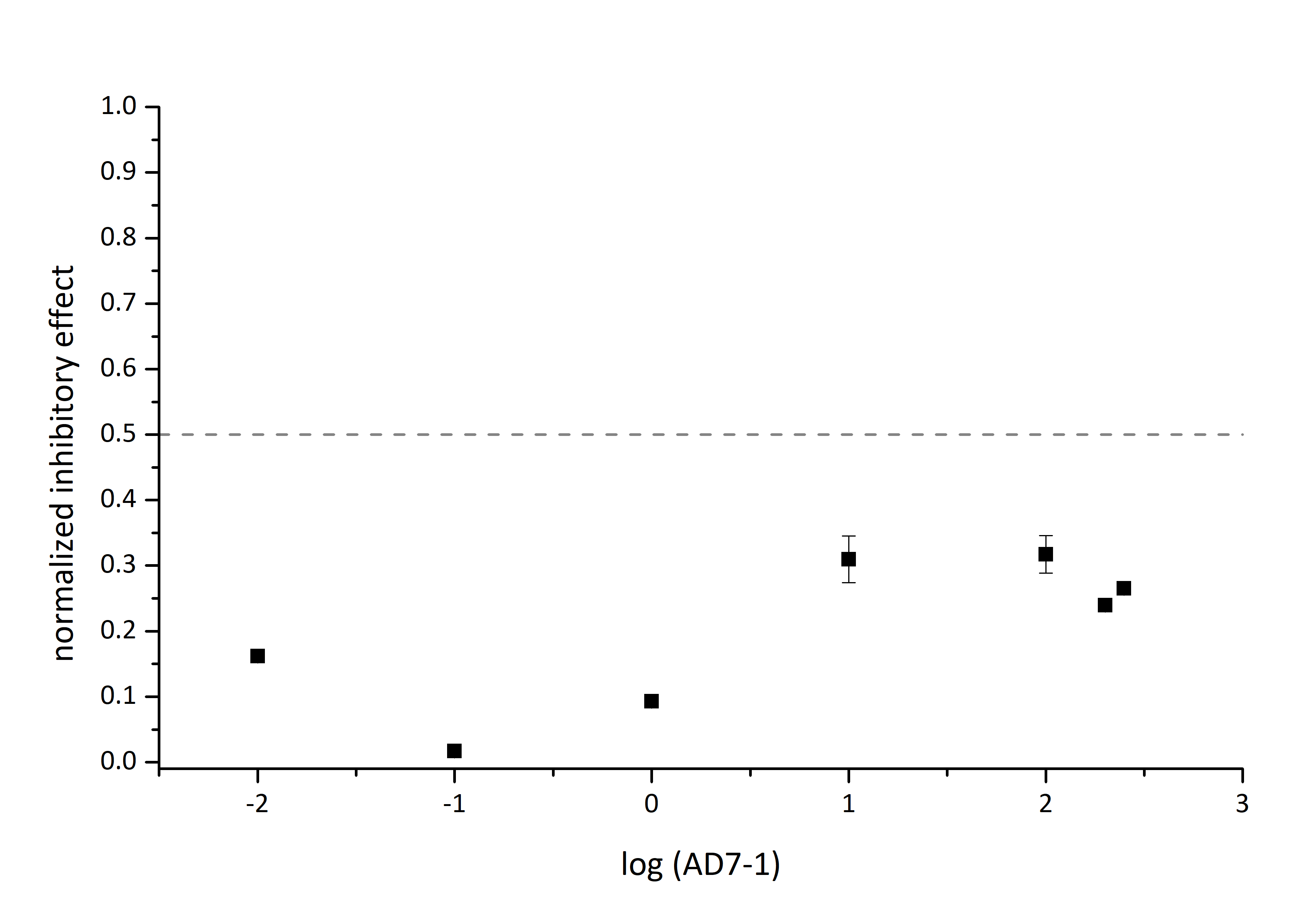


*y = 0.5*

*y = 0.5*

*y = 0.5*

*y = 0.5*

AD7-1 K_i_^app^ > 250µM

AD7-1* K_i_^app^ > 250µM

AD3-11 K_i_^app^ > 50µM

AurD K_i_^app^ > 10µM


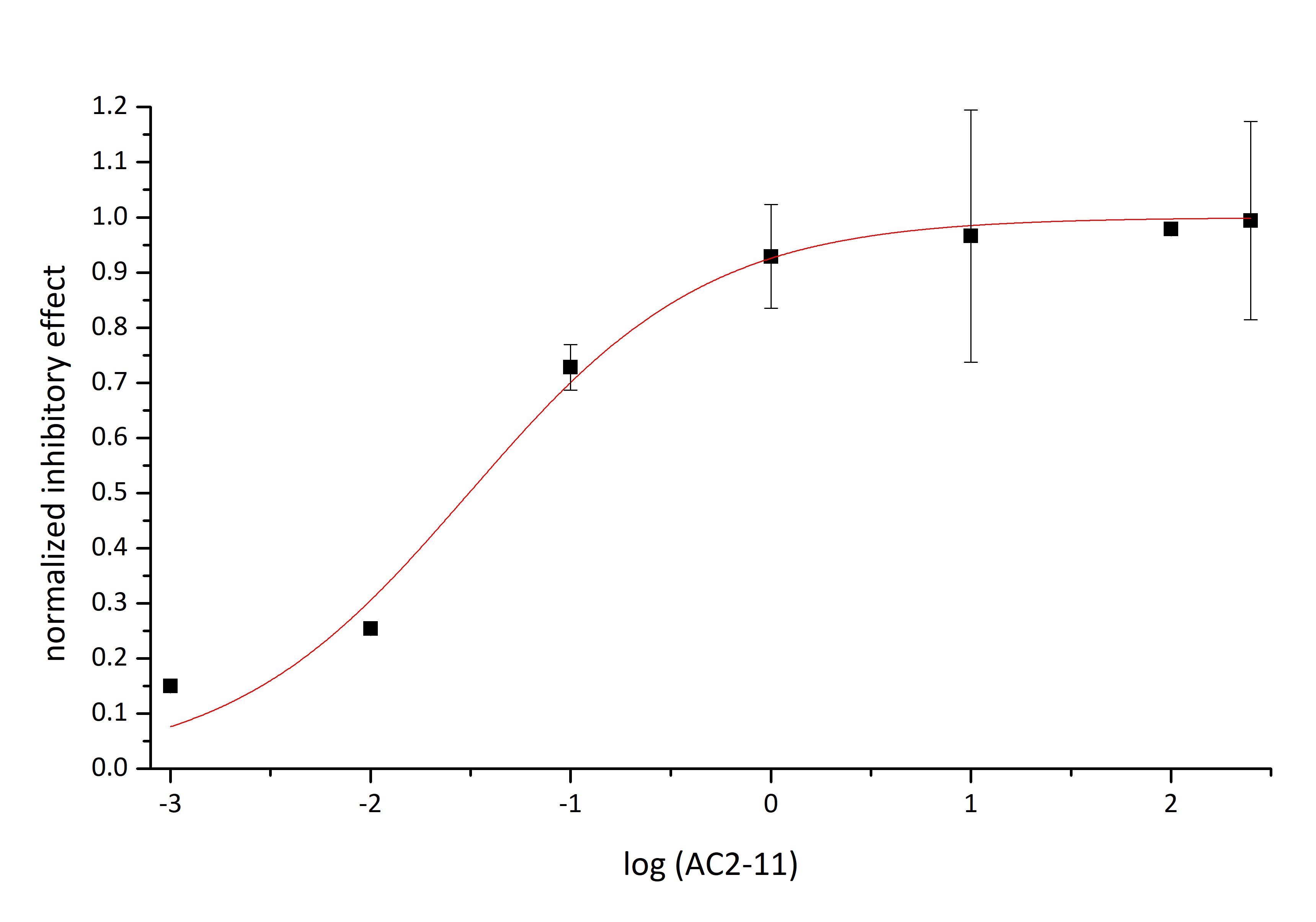

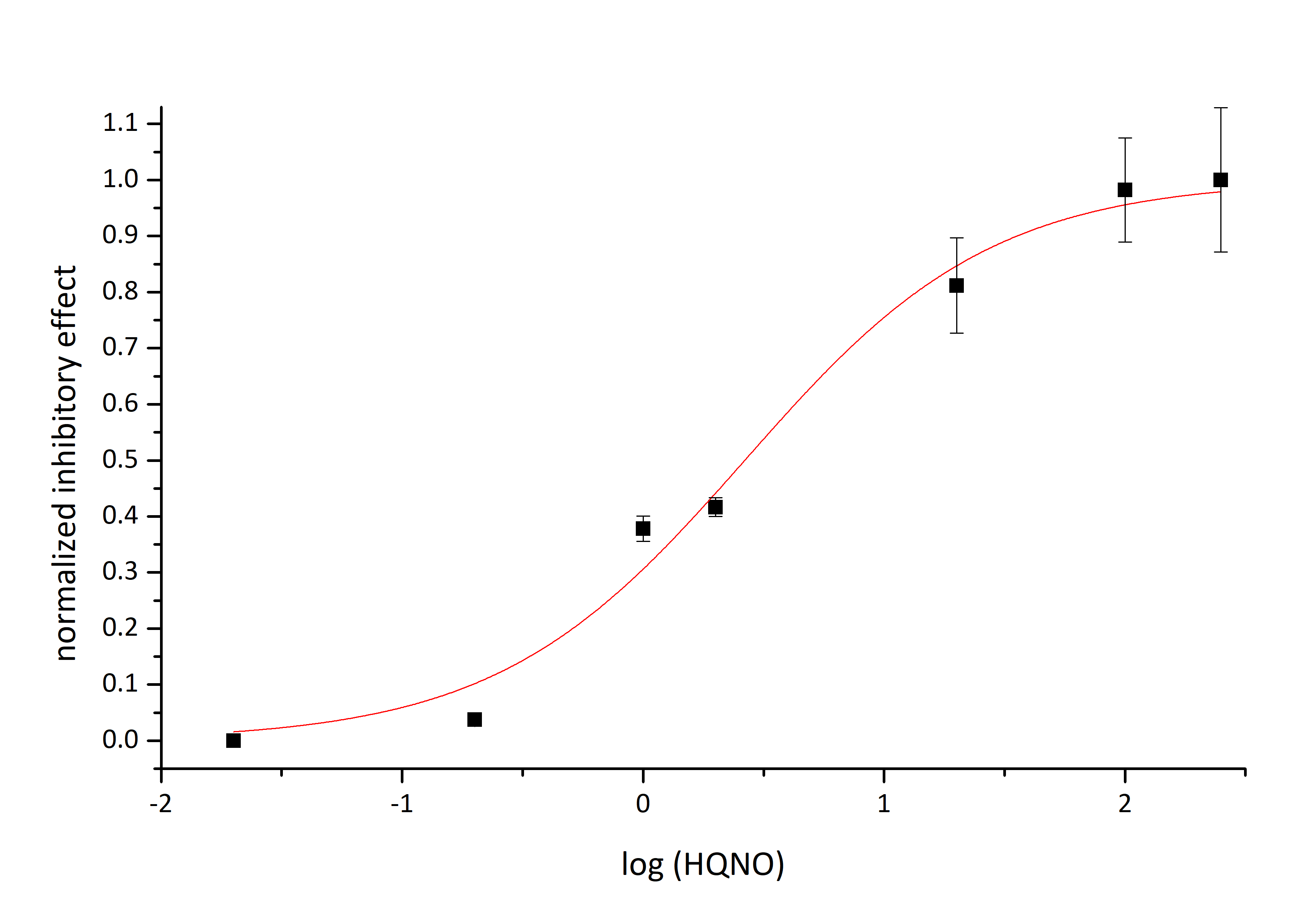


HQNO K_i_^app^ 2.6 ± 0.4µM

AC2-11 K_i_^app^ 0.031 ± 0.006µM

**Figure S-5**: Half- logarithmic dose response curves for *E. coli* cytochrome ***bo*_3_** oxidase. K_i_^app^ determined by functional oxygen reductase assay. 30nM ***bo*_3_** in presence up to 250µM inhibitor. Each point represents mean ± S.E.M. (n=3). Sigmoidal fit (red line) with DoseResp fit (Origin LabPro9.5).

## Synthesis

The experimental procedures for the bromination of allyl alcohols, the substitution of ethyl acetoacetate with the obtained allyl bromides, and the preparation of the corresponding enamines and, finally the Conrad-Limpach cyclization to obtain the substituted aurachin D derivatives were performed as described in the publication of *Dejon et al.* ^1^

**General procedure for the synthesis of allyl bromides from allyl alcohols**

Mesyl chloride (MsCl) (1mmol, 1.3eq.) as well as triethylamine (ET_3_N) (1mmol, 2eq.) were successively added dropwise to a -40°C cooled solution of allyl alcohol (1 mmol, 1.0eq) in dry THF (1.5ml). The reaction mixture was then stirred under argon atmosphere for one hour at the same temperature. Subsequently, the reaction was quenched by adding LiBr (4mmol, 4eq.) before the suspension was stirred at 0°C for another hour. The suspension was then diluted with ice-cold water and extracted three times with chilled diethyl ether, which was then washed twice with sodium hydrogen carbonate and brine respectively. The solution was then dried with MgSO_4_ and the solvent was removed under vacuum. The obtained allyl bromide was then used for further steps without further purification.

**General procedure for the substitution of ethyl acetoacetate with synthesized allyl bromides**

Ethyl acetoacetate (1mmol, 1.0eq.) was added dropwise to a suspension of sodium hydride (60% in oil, 1.1 mmol) in anhydrous THF cooled to 0°C. After stirring at 0°C for 20min, a solution of (1.1mmol, 1.1eq.) in dry THF (250µl) was added and the reaction mixture was stirred overnight at room temperature. The crude product was washed with water and brine, extracted twice with diethyl ether, and purified by column chromatography (Cy. hexane/ethyl acetate 5:1)

**General procedure for the synthesis of aurachin derivatives**

**Step 1: Synthesis of enamine intermediates**

Aniline (1mmol, 1eq.) and catalytic amount of ceric ammonium nitrate were added to the previously synthesized 2-allyl acetoacetate (1mmol, 1eq.) dissolved in abs. ethanol (2ml). The obtained mixture was stirred at room temperature for 1h. It was then washed three times in succession with water and brine, extracted with dichloromethane and dried over MgSO_4_. Evaporation of the solvent yielded the desired enamine, which was used in the cyclization step.

**Step 2: Conrad-Limpach cyclization to aurachin derivatives**

The synthesized enamines were rapidly added to diphenyl ether heated to 250°C without further purification and stirred for 30min. The precipitated white solid contains diphenyl ether, which was removed by chromatographic separation with cyclohexane and ethyl acetate (9:1, 6:1, 3:1).


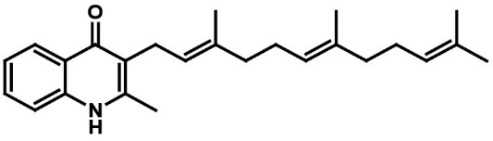
**Aurachin-D, ^1^H NMR** (DMSO-d_6_, 400 MHz): *δ* = 11.41 (br, 1H), 8.11 (d, ^3^*J*(H,H) = 8.0 Hz, 1H), 7.58 (td, ^3^*J*(H,H) = 6.7 Hz, ^4^*J*(H,H) = 1.3 Hz, 1 H), 7.48 (d, ^3^*J*(H,H) = 8.2 Hz, 1H), 7.25 (td, ^3^*J*(H,H) = 6.6 Hz, ^4^*J*(H,H) = 1.1 Hz 1H), 5.03 (m, 3H), 3.24 (d, ^3^*J*(H,H) = 6.6 Hz, 2H), 2.35 (s, 3H), 1.86-2.06 (m, 8H), 1.74 (s, 3H), 1.61 (s, 3H), 1.53(s, 1H), 1.51 (s, 1H) ppm. **^13^C NMR:** (DMSO-d_6_, 100.6 MHz): *δ* = 175.2, 146.2, 146.1, 139.1, 134.3, 134.2, 133.6, 133.5, 130.8, 130.8, 130.8, 130.5, 125.1, 124.9, 124.1, 123.8, 123.4, 123.0, 123.0, 122.3, 118.2, 117.4 ppm. **MS (ESI):** m/z calcd for C_25_H_33_NO: 362.26 [*M* - H] ^-^; found 362.30. **Purity grade**: 98%, **TLC**-**Rf** (CyH:EE,3:1): 55%.


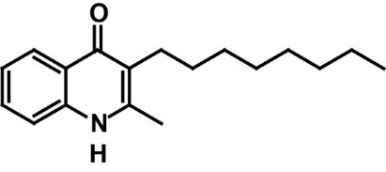
**AD8-1, ^1^H NMR** (DMSO-d_6_, 400 MHz): *δ* = 11.35 (br, 1H), 8.04 (d, ^3^*J*(H,H) = 7.7 Hz, 1H), 7.57 (td, ^3^*J*(H,H) = 7.6 Hz, ^4^*J*(H,H) = 1.3 Hz, 1 H), 7.46 (d, ^3^*J*(H,H) = 8.3 Hz, 1H), 7.24 (t, ^3^*J*(H,H) = 7.6 Hz, 1H), 2.48 (t, ^3^*J*(H,H) = 7.5 Hz, 2H), 2.39 (s, 3H), 1.20 – 1.44 (m, 12H), 0.86 (t, ^3^*J*(H,H) = 6.6 Hz, 3H), ppm. **MS (ESI):** m/z calcd for C_18_H_25_NO: 270.19 [*M* - H] ^-^; found 270.34. **Purity grade**: 99%, **TLC**-**Rf** (CyH:EE,3:1): 44%.


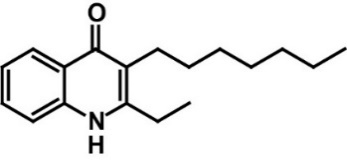
**AD7-2, ^1^H NMR** (DMSO-d_6_, 400 MHz): *δ* = 11.26 (br, 1H), 8.04 (d, ^3^*J*(H,H) = 8.0 Hz, 1H), 7.56 (tt, ^3^*J*(H,H) = 7.6 Hz, ^4^*J*(H,H) = 1.5 Hz, 1 H), 7.49 (d, ^3^*J*(H,H) = 8.6 Hz, 1H), 7.21-7.25 (tt, ^3^*J*(H,H) = 7.5 Hz, ^4^*J*(H,H) = 1.5 Hz, 1H), 2.68 (q, ^3^*J*(H,H) = 8.3 Hz, 2H), 1.20 – 1.44 (m, 12H), 0.86 (t, ^3^*J*(H,H) = 7.1 Hz, 4H) ppm. **MS (ESI):** m/z calcd for C_18_H_25_NO: 271.19 [*M* - H] ^-^; found 270.30. **Purity grade**: 96%, **TLC**-**Rf** (CyH:EE,3:1): 59%.


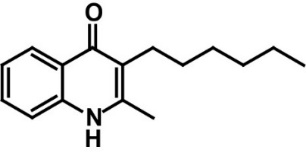
**AD6-1, ^1^H NMR** (DMSO-d_6_, 250 MHz): *δ* = 11.4 (br, 1H), 8.04 (dd, ^3^*J*(H,H) = 8.1 Hz, ^4^*J*(H,H) = 1.2 Hz, 1H), 7.60 (td, ^3^*J*(H,H) = 7.6 Hz, ^4^*J*(H,H) = 1.5 Hz, 1 H), 7.5 (d, ^3^*J*(H,H) = 7.9 Hz, 1H), 7.28 (tt, ^3^*J*(H,H) = 7.4 Hz, ^4^*J*(H,H) = 1.1 Hz, 1H), 2.43 (s, 3H), 1.28 – 1.46 (m, 7H), 0.86-0.94 (m, ^3^H) ppm. **MS (ESI):** m/z calcd for C_16_H_21_NO: 243.16 [*M* - H] ^-^; found 244.22. **Purity grade**: 98%, **TLC**-**Rf** (CyH:EE,3:1): 50%.


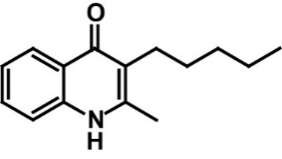
**AD5-1, ^1^H NMR** (DMSO-d_6_, 250 MHz): *δ* = 11.35 (br, 1H), 8.04 (dd, ^3^*J*(H,H) = 8.1 Hz, ^4^*J*(H,H) = 1.2 Hz, 1H), 7.57 (td, ^3^*J*(H,H) = 7.6 Hz, ^4^*J*(H,H) = 1.5 Hz, 1 H), 7.50 (d, ^3^*J*(H,H) = 8.0 Hz, 1H), 7.24 (tt, ^3^*J*(H,H) = 7.4 Hz, ^4^*J*(H,H) = 1.2 Hz, 1H), 2.39 (s, 3H), 1.26 – 1.52 (m, 6H), 0.88(t, ^3^*J*(H,H) = 6.3 Hz) ppm. **MS (ESI):** m/z calcd for C_15_H_19_NO: 229.15 [*M* - H] ^-^; found 230.20. **Purity grade**: 98.5%, **TLC**-**Rf** (CyH:EE,3:1): 48%.


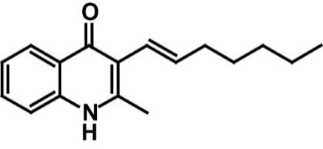
**AD7-1*, ^1^H NMR** (DMSO-d_6_, 400 MHz): *δ* = 11.27 (br, 1H), 8.04 (d, ^3^*J*(H,H) = 8.0 Hz, 1H), 7.57 (tt, ^3^*J*(H,H) = 7.6 Hz, ^4^*J*(H,H) = 1.5 Hz, 1 H), 7.49 (d, ^3^*J*(H,H) = 8.6 Hz, 1H), 7.23 (tt, ^3^*J*(H,H) = 7.5 Hz, ^4^*J*(H,H) = 1.5 Hz, 1H), 2.68 (q, ^3^*J*(H,H) = 8.3 Hz, 2H), 1.20 – 1.44 (m, 12H), 0.86 (t, ^3^*J*(H,H) = 7.1 Hz, 4H) ppm.
**MS (ESI):** m/z calcd for C_18_H_25_NO: 254.16. **Purity grade**: 99.5%, **TLC**-**Rf** (CyH:EE,3:1): 53%.


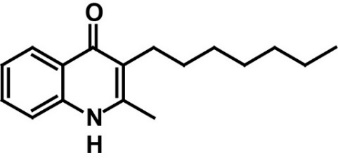
**AD7-1, ^1^H NMR** (DMSO-d_6_, 500 MHz): *δ* = 11.35 (br, 1H), 8.04 (d, ^3^*J*(H,H) = 8.3 Hz, 1H), 7.56 (td, ^3^*J*(H,H) = 7.6 Hz, ^4^*J*(H,H) = 1.5 Hz, 1 H), 7.46 (d, ^3^*J*(H,H) = 8.1 Hz, 1H), 7.23 (t, ^3^*J*(H,H) = 7.5 Hz, 1H), 2.48 (t, ^3^*J*(H,H) = 7.5 Hz, 2H), 2.1 (s, 3H), 1.20 – 1.44 (m, 9H), 0.86 (t, ^3^*J*(H,H) = 6.6 Hz, 3H), ppm. **^13^C NMR:** (DMSO-d_6_, 100.6 MHz): *δ* = 176.0, 170.8, 146.1, 139.6, 131.3, 125.6, 123.8, 122.7. 119.6, 117.9, 62.1, 31.7, 25.5, 25.3, 21.3, 18.0, 17.9 ppm. **MS (ESI):** m/z calcd for C_187_H_23_NO: 256.2 [*M* - H] ^-^; found 256.34.
**Purity grade**: 99.5%, **TLC**-**Rf** (CyH:EE,3:1): 50%.


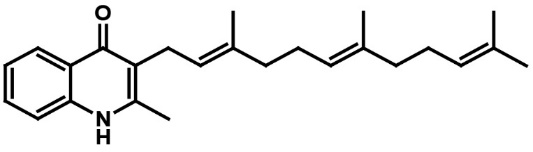
**
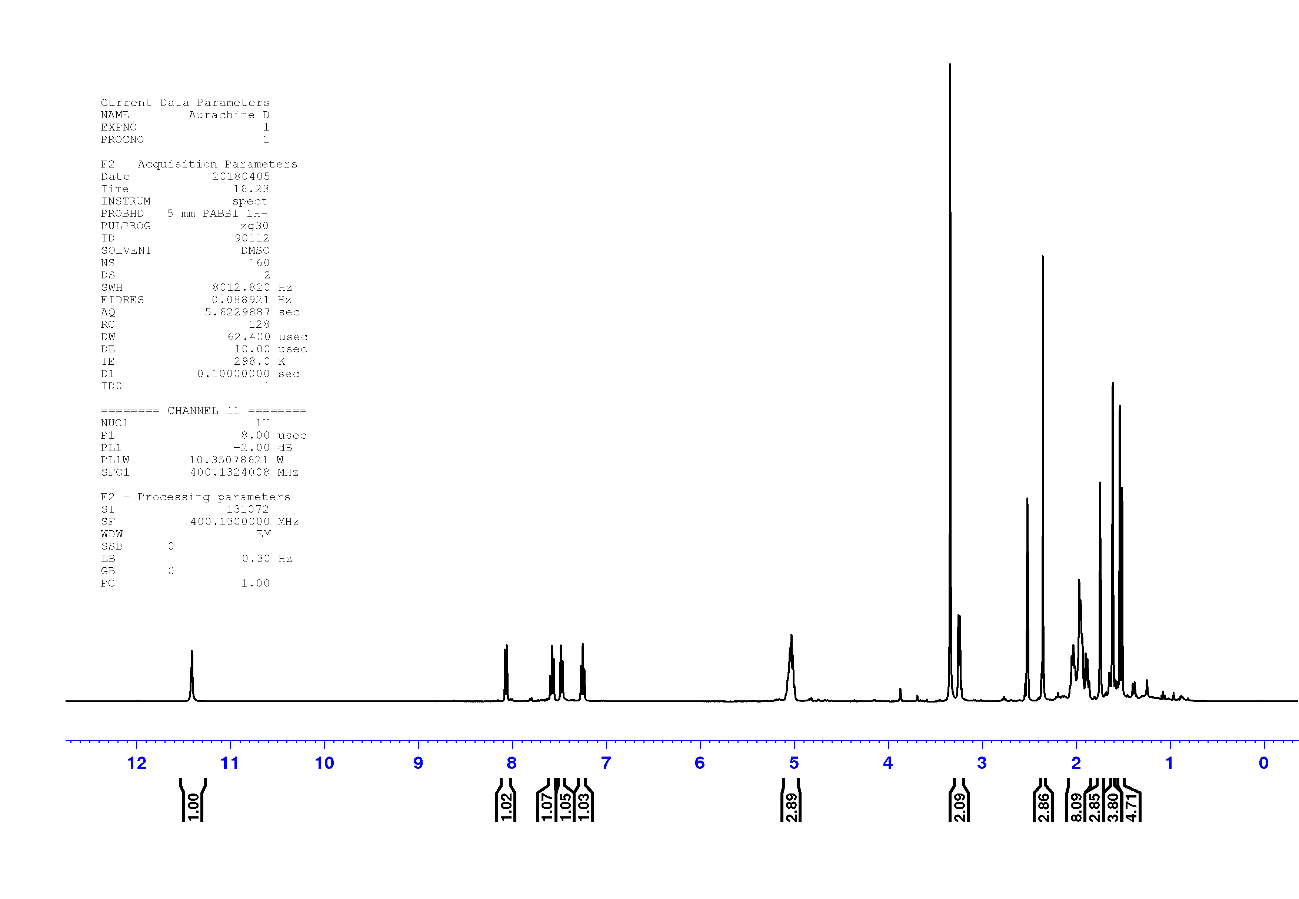
Aurachin D**


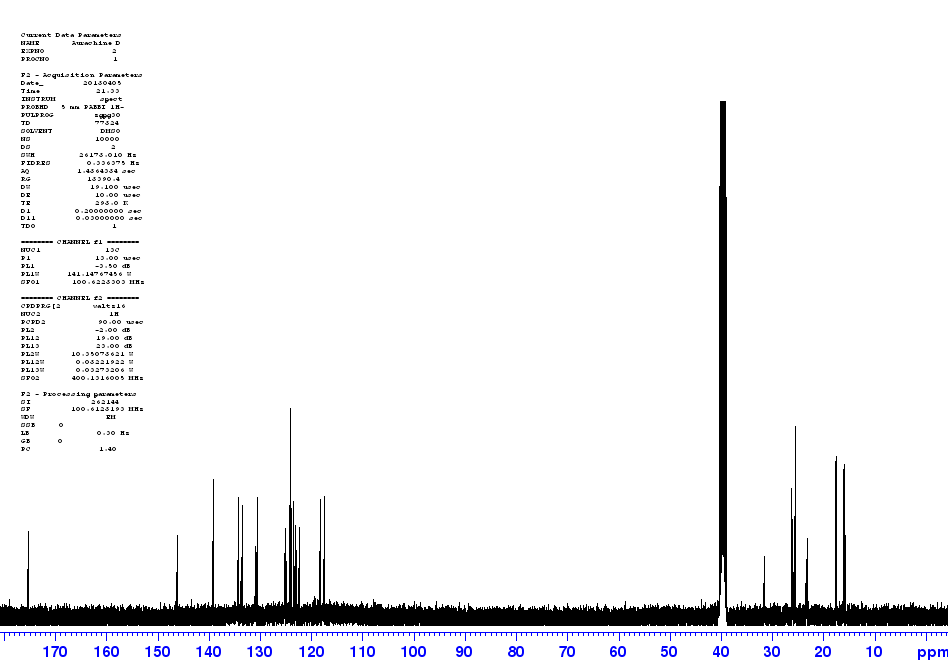


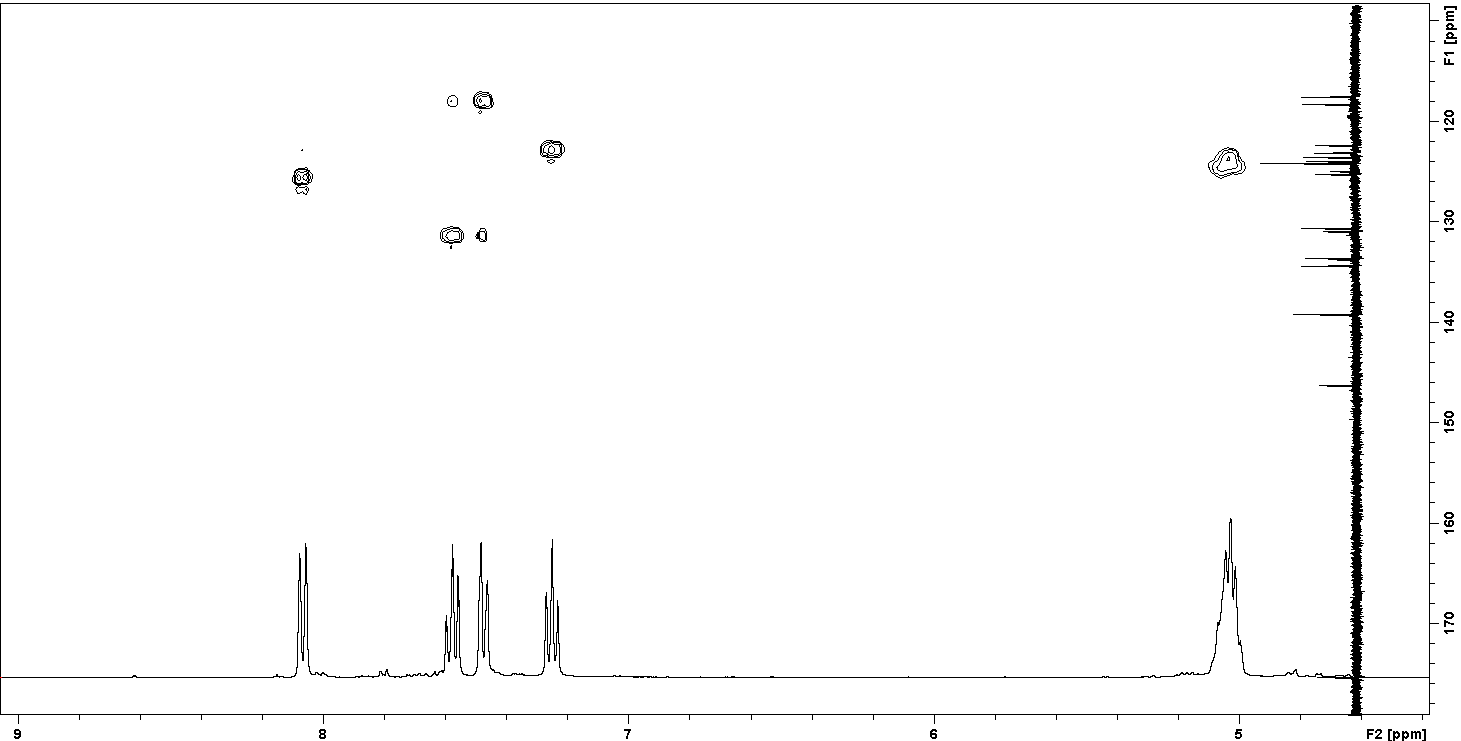


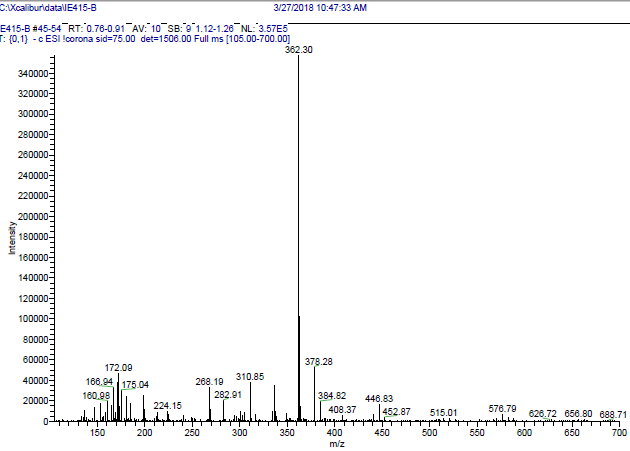


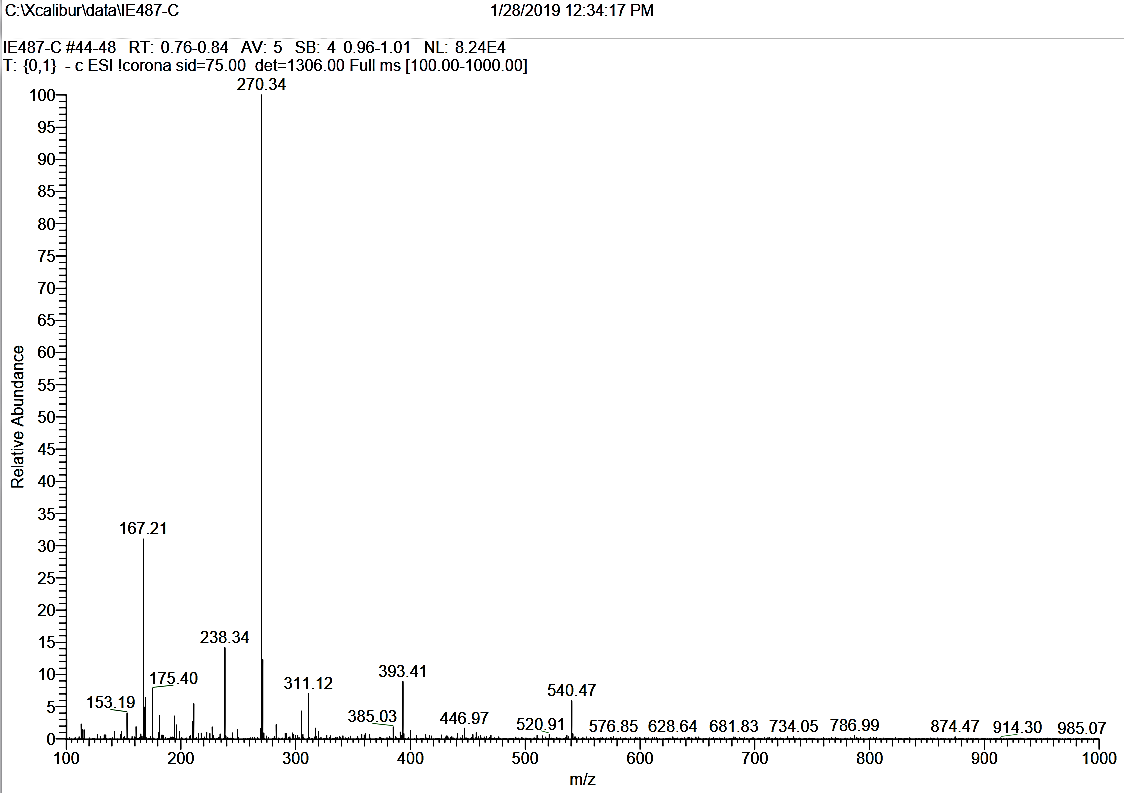

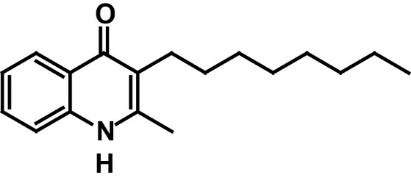

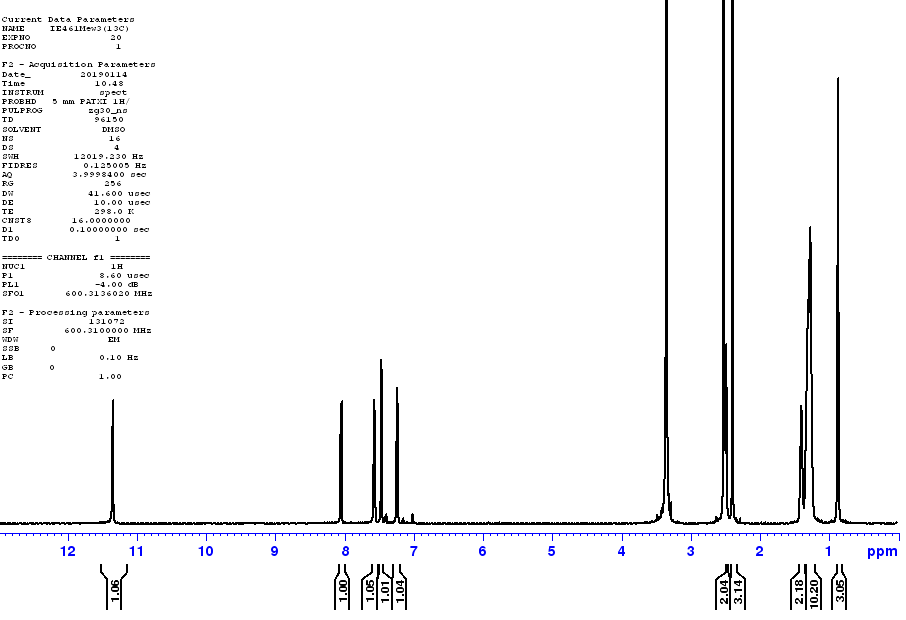
**AD8-1**


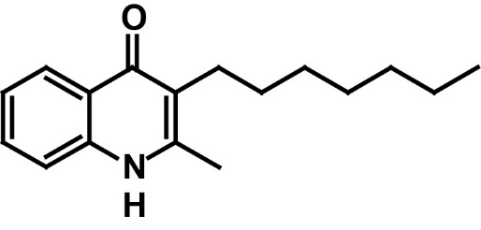

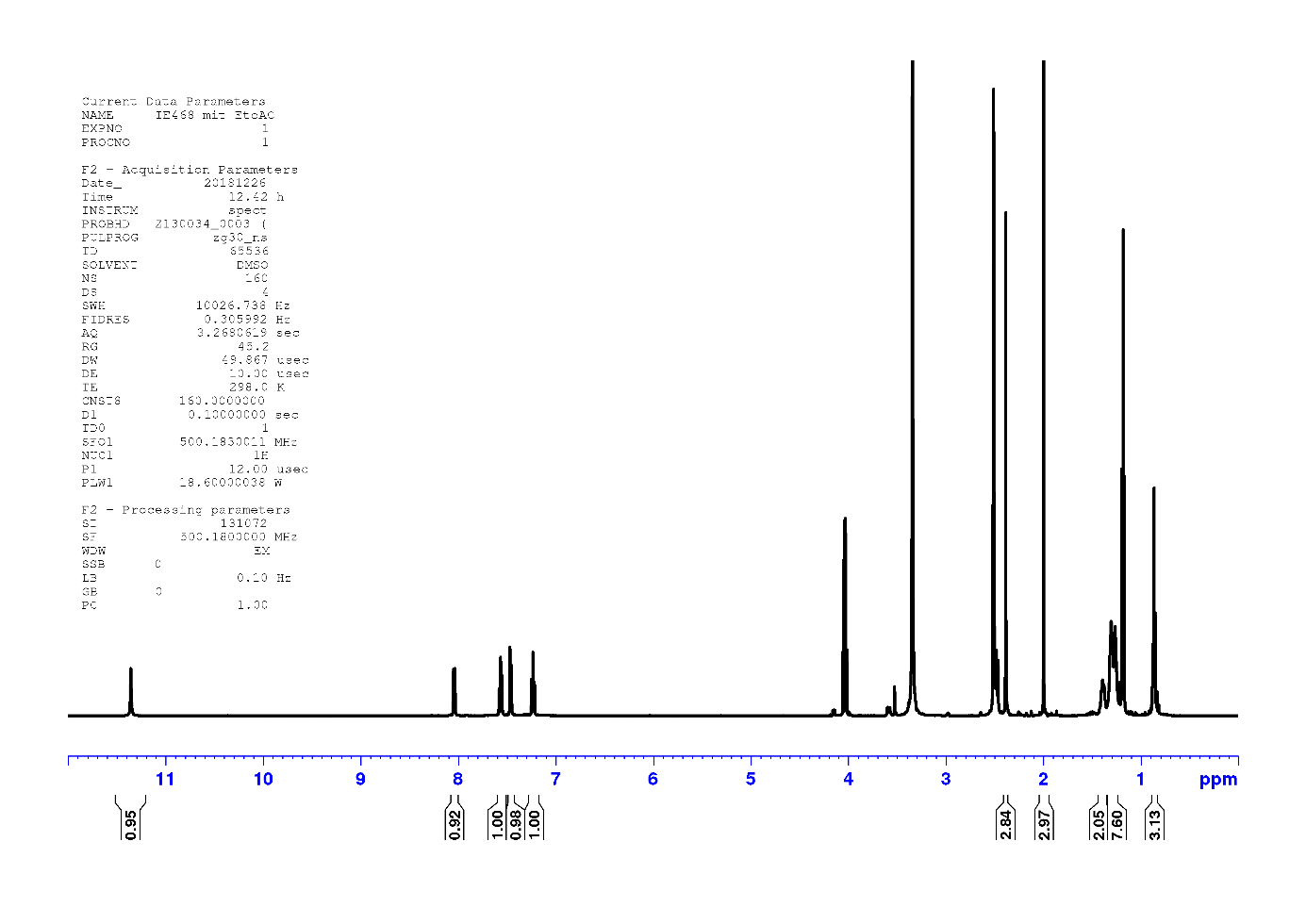
**AD7-1**


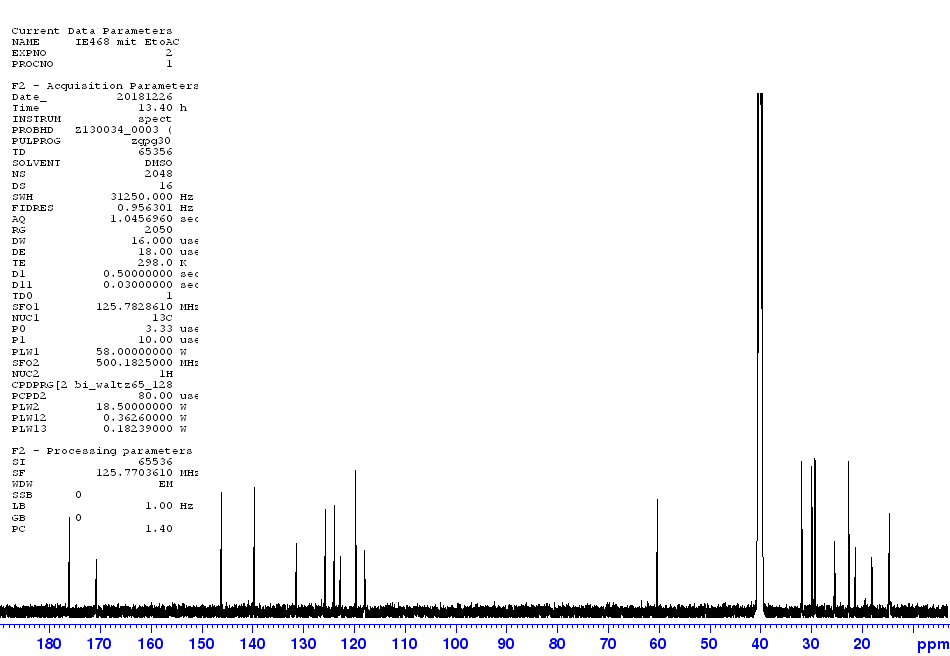


**AD7-2**


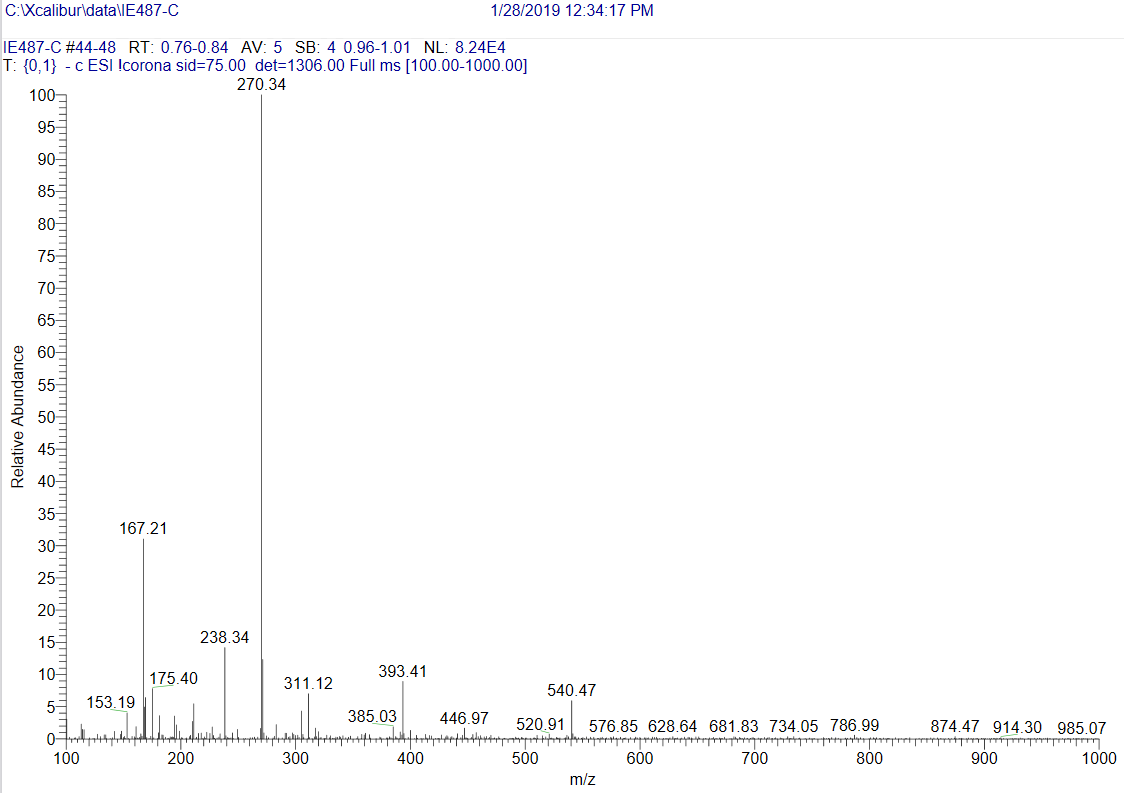

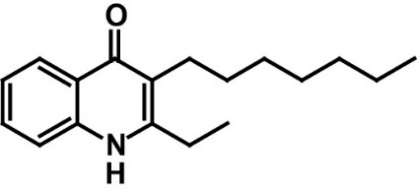
**
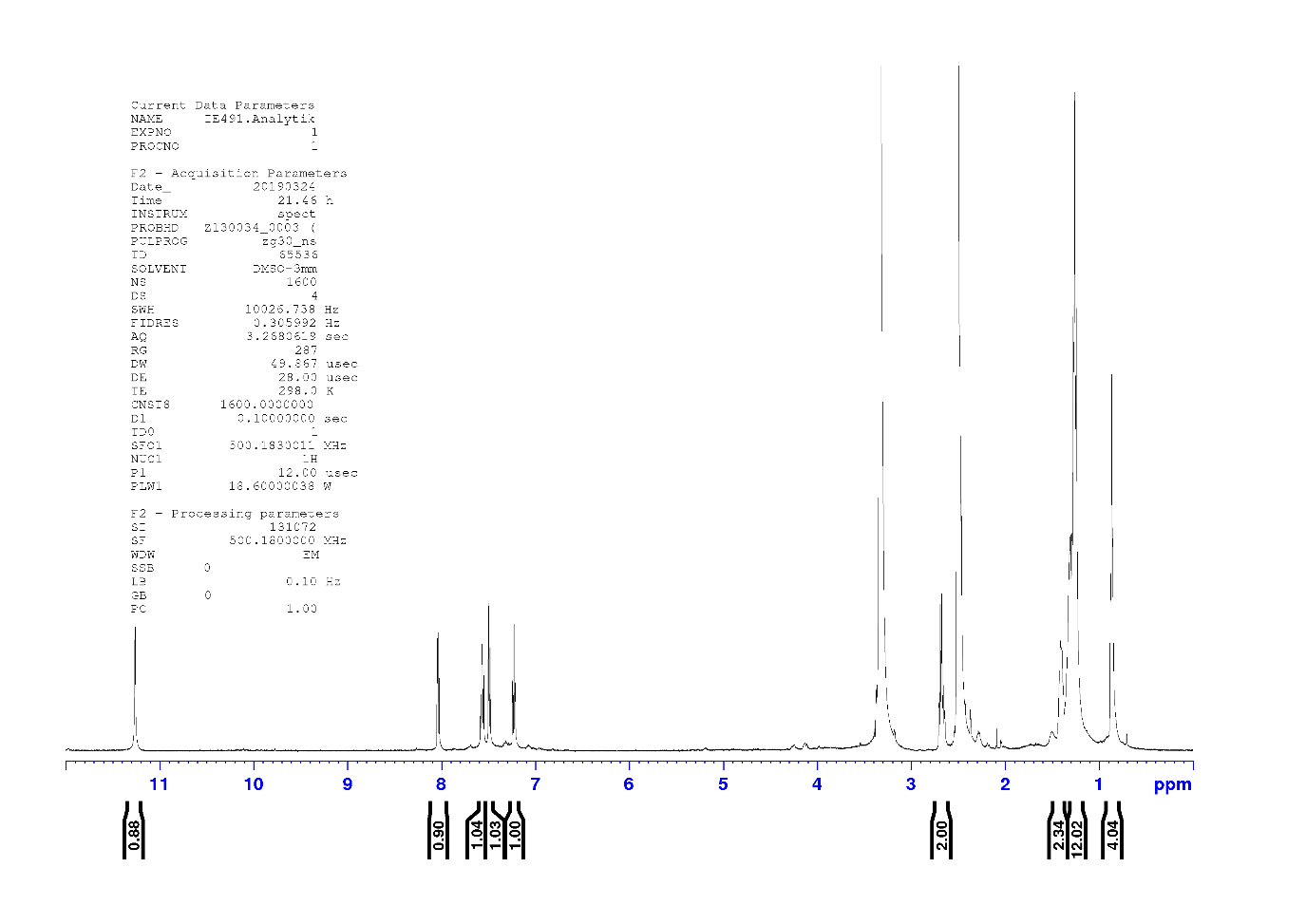
**

**AD6-1**


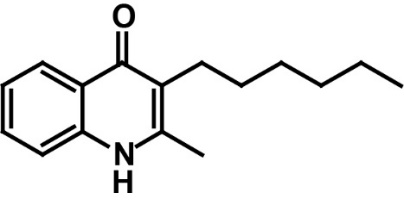

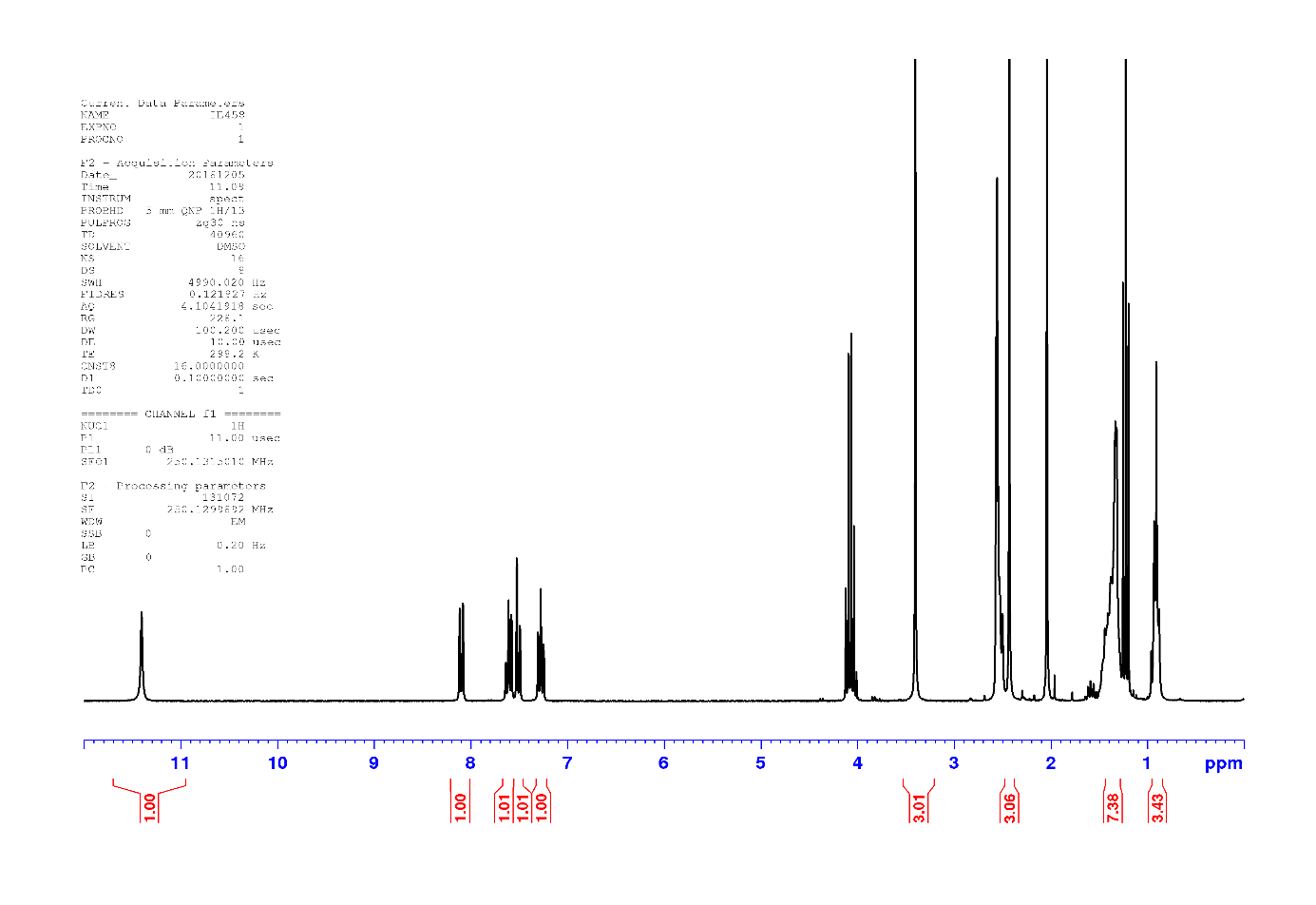


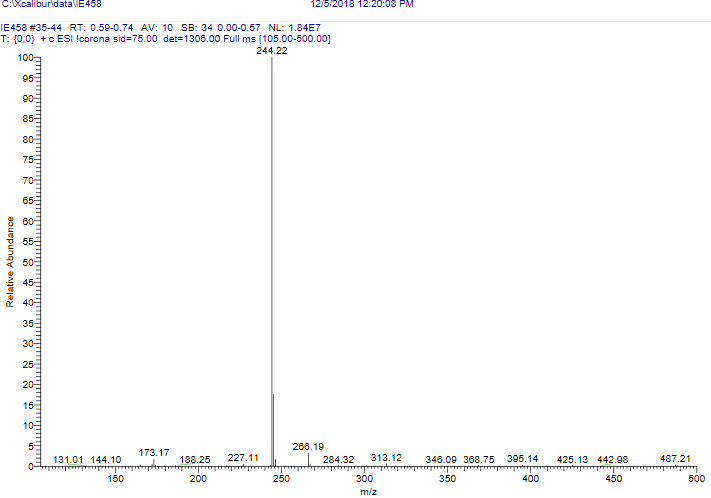


**AD5-1**
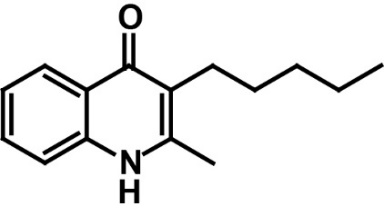


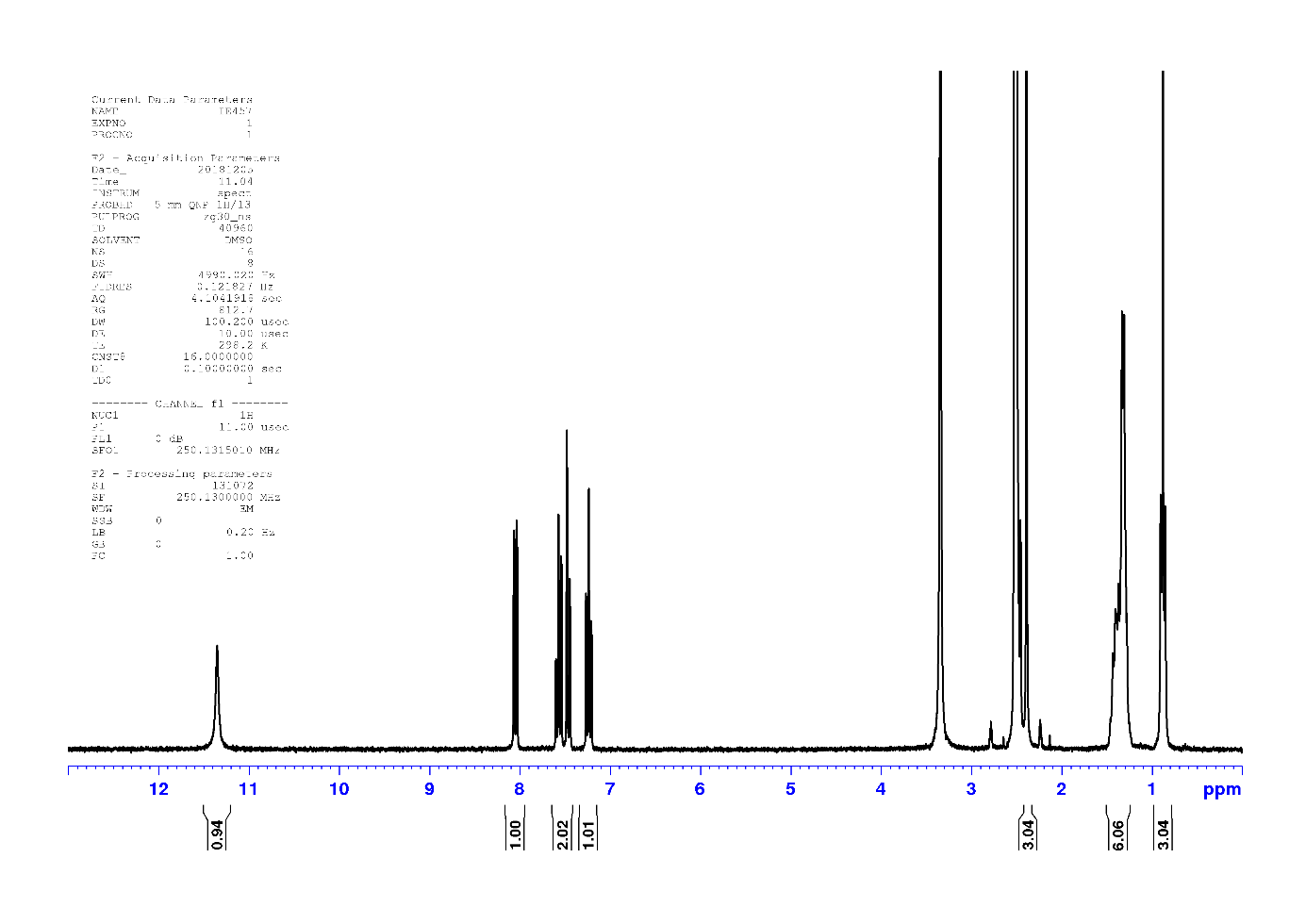


**
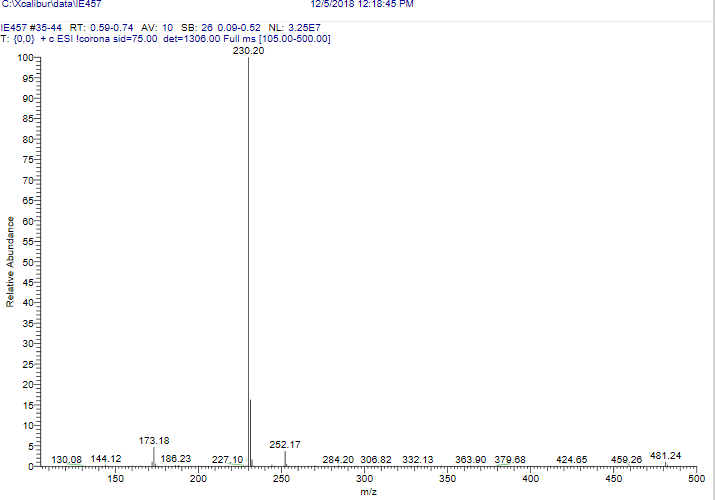
**


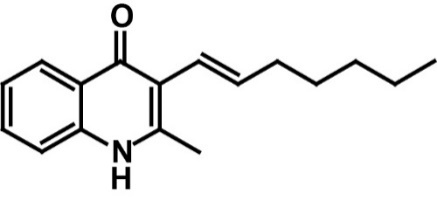

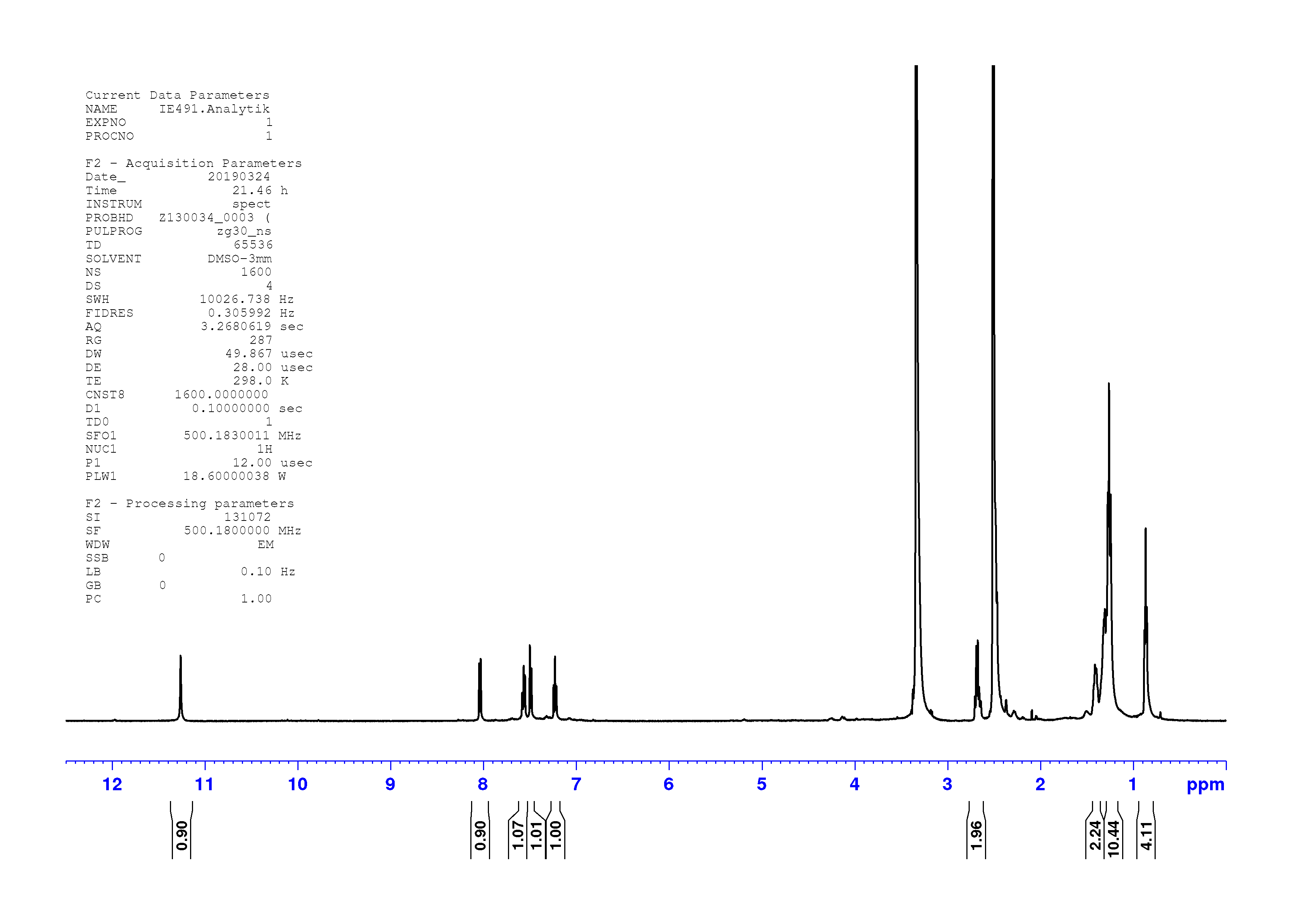
**AD7-1***

## References

## 1. Dejon, L. & Speicher, A. Synthesis of aurachin D and isoprenoid analogues from the myxobacterium Stigmatella aurantiaca. *Tetrahedron Lett.* 54, 6700–6702 (2013).
